# CST6 suppresses osteolytic bone disease in multiple myeloma by blocking osteoclast differentiation

**DOI:** 10.1172/JCI159527

**Published:** 2022-09-15

**Authors:** Dongzheng Gai, Jin-Ran Chen, James P. Stewart, Intawat Nookaew, Hasem Habelhah, Cody Ashby, Fumou Sun, Yan Cheng, Can Li, Hongwei Xu, Bailu Peng, Tarun K. Garg, Carolina Schinke, Sharmilan Thanendrarajan, Maurizio Zangari, Fangping Chen, Bart Barlogie, Frits van Rhee, Guido Tricot, John D. Shaughnessy, Fenghuang Zhan

**Affiliations:** 1 Myeloma Center, Winthrop P. Rockefeller Cancer Institute, Department of Internal Medicine, University of Arkansas for Medical Sciences, Little Rock, Arkansas, USA.; 2 Department of Hematology, Xiangya Hospital, Central South University, Changsha, Hunan, China.; 3 Arkansas Children’s Nutrition Center and; 4 Department of Biomedical Informatics, College of Medicine, University of Arkansas for Medical Sciences, Little Rock, Arkansas, USA.; 5 Department of Pathology, Carver College of Medicine, University of Iowa, Iowa City, Iowa, USA.

**Keywords:** Bone Biology, Hematology, Bone disease, Cancer, Osteoclast/osteoblast biology

## Abstract

Osteolytic bone disease is a hallmark of multiple myeloma (MM). A significant fraction (~20%) of MM patients do not develop osteolytic lesions (OLs). The molecular basis for the absence of bone disease in MM is not understood. We combined PET-CT and gene expression profiling (GEP) of purified BM CD138^+^ MM cells from 512 newly diagnosed MM patients to reveal that elevated expression of cystatin M/E (*CST6*) was significantly associated with the absence of OL in MM. An enzyme-linked immunosorbent assay revealed a strong correlation between CST6 levels in BM serum/plasma and *CST6* mRNA expression. Both recombinant CST6 protein and BM serum from patients with high CST6 significantly inhibited the activity of the osteoclast-specific protease cathepsin K and blocked osteoclast differentiation and function. Recombinant CST6 inhibited bone destruction in ex vivo and in vivo myeloma models. Single-cell RNA-Seq showed that CST6 attenuates polarization of monocytes to osteoclast precursors. Furthermore, CST6 protein blocks osteoclast differentiation by suppressing cathepsin-mediated cleavage of NF-κB/p100 and TRAF3 following RANKL stimulation. Secretion by MM cells of CST6, an inhibitor of osteoclast differentiation and function, suppresses osteolytic bone disease in MM and probably other diseases associated with osteoclast-mediated bone loss.

## Introduction

Osteolytic lesions (OLs) of the axial skeleton are a hallmark of multiple myeloma (MM), a malignancy of antibody-secreting plasma cells (PCs). While bone metastases are seen in many cancers, the presence of OLs is one of the diagnostic criteria for MM. Osteolysis in MM is linked to both suppressed osteoblastogenesis and increased osteoclastogenesis (1). New bone formation is suppressed, at least in part, via Dickkopf-1 (DKK-1) mediated inhibition of Wnt/β-catenin signaling, which is essential for osteoblast differentiation (2). DKK1 also increases osteoclast numbers by increasing the RANKL/osteoprotegerin (OPG) ratios in the BM microenvironment (3–5).

Using global gene expression profiling (GEP), we and others have created a molecular classification of MM (6–8). Correlation of clinical parameters with molecular subtypes revealed a statistically significant lower incidence of bone disease in a subtype of disease we referred to as the low-bone (LB) disease subtype (6). The existence of this subgroup was independently verified (6–8). These data strongly suggest that MM lacking bone disease represents a distinct pathologic entity.

Proteostasis or protein homeostasis is a process that regulates intracellular proteins to maintain a balanced, functional proteome (9). Protease-mediated hydrolysis plays a key role in maintaining proteostasis. Several important proteases have been identified in different organelles; the proteasome, cathepsins, human caseinolytic protease p (hCIpP), and metallopeptidases (MMPs) are in the cytoplasm, lysosome, mitochondria, and extracellular environment, respectively. The intracellular proteases include the cytoplasmic ubiquitin-proteasome system (UPS) and autophagy lysosomal system (ALS), which regulate intracellular protein degradation and also osteoclast differentiation and function (10). Osteoclasts are multinucleated cells formed by the fusion of mononuclear progenitors of the monocyte/macrophage lineage (11). RANKL interacts with its cognate receptor RANK in the presence of macrophage CSF (M-CSF) to promote osteoclast differentiation and maturation via the NF-κB signaling pathway. Both canonical and noncanonical NF-κB pathways are regulated by the UPS during osteoclastogenesis (12). The canonical signaling pathway is activated within a short period of time, leading to IκBα degradation and translocation of p65/p50 heterodimers into the nucleus (13). In the noncanonical signal pathway, RANKL stimulation induces TRAF3 degradation, resulting in the stabilization of NF-κB–inducing kinase (NIK). NIK activates IκB kinase α (IKKα), which promotes p100 processing to p52 and the subsequent nuclear translocation of RelB/p52 complexes (12, 13). It is known that p100 is a suppressor of the noncanonical NF-κB pathway in osteoclastogenesis (14). The p100 can be removed from the cytosol either through processing or complete degradation triggered by the NIK/IKK1 complex (15). However, it is unknown whether ALS or other proteases are involved in p100 protein processing.

A small but significant fraction (~20%) of patients with MM present without OL at diagnosis. The molecular basis for the absence of OL in MM is currently not understood. PET-CT is recommended by the International Myeloma Working Group (IMWG) to ascertain the presence of MM focal bone lesions (16). In the current study, we combined PET-CT with global GEP of CD138-selected PCs from the BM of 512 patients with newly diagnosed MM (NDMM) to identify secreted molecules that might account for the absence of OL in MM. This analysis identified a marked link between the absence of PET-CT–defined OL and elevated expression of the soluble protease inhibitor *CST6*. CST6, a 14 to 17 kDa secretory protein, is a member of the family of type 2 cystatins, cysteine proteinase inhibitors that regulate lysosomal cysteine proteases and the asparaginyl endopeptidase legumain (*LGMN*). We have demonstrated that both purified CST6 and BM serum from patients with high CST6 expression suppress osteoclast function and differentiation in a CST6-dependent fashion and that recombinant CST6 inhibits bone disease in an in vivo myeloma mouse model. Mechanistic studies reveal that CST6 regulates osteoclastogenesis through at least 3 different mechanisms: depolarization of osteoclast precursors (OCPs), stabilization of p100 and TRAF3, and inhibition of the extracellular environmental protease cathepsin K (CTSK).

## Results

### Elevated expression of CST6 is linked to absence of MM bone disease.

**Figure 1:**
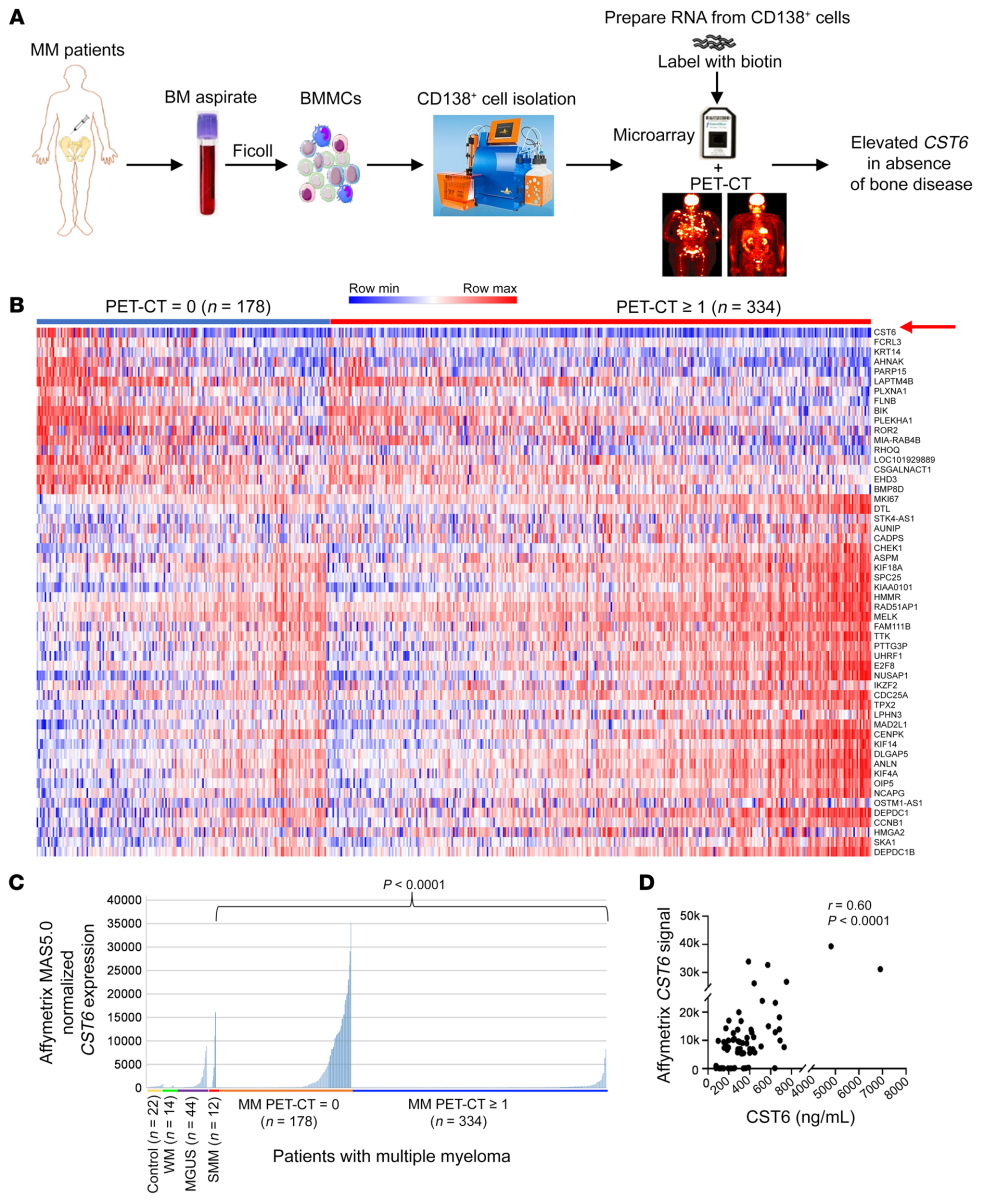
High expression of *CST6* is linked to the absence of bone lesions in MM. (**A**) Workflow of the study. BMMC, BM mononuclear cells. (**B**) Heatmap showing that 54 genes were significantly differentially expressed in MM cells from patients with no (*n* = 178) or 1 or more focal lesions (*n* = 334) on PET-CT (*P* < 0.0001). Shown are 17 genes with elevated levels of expression in MM cells from patients with no lesions on PET-CT and ranked from top to bottom by significance; and 37 genes with significantly upregulated expression in tumor cells with 1 or more lesions on PET-CT and ranked from bottom to top by significance. Gene symbols are listed on the right. (**C**) Affymetrix MAS5.0 normalized mRNA expression signal is indicated on the *y* axis. Expression level of *CST6* in each sample is indicated by the height of the bar. Samples are ordered from the lowest to highest level of expression of *CST6* from left to right on the *x* axis. *P* value was obtained using 2-tailed, unpaired Student’s *t* test. (**D**) Dot plot shows the correlation between *CST6* mRNA in purified MM tumor cells and BM serum CST6 levels. The level of expression of *CST6* mRNA was quantified by microarray analysis, and CST6 protein was measured by ELISA in 75 NDMM patients. Each spot indicates the relative relation of *CST6* mRNA and protein expression levels for a patient. There was a significant correlation between the level of *CST6* mRNA in MM cells and the level of CST6 protein in MM BM serum/plasma (*r* = 0.60, *P* < 0.0001). *P* value was obtained by Pearson’s correlation and a linear regression analysis.

We correlated global mRNA expression levels in CD138-selected BM PCs from 512 NDMM patients with the presence or absence of PET-CT–defined focal OL (Figure 1A). Of these, 178 had no evidence of PET-CT bone lesions, while 334 cases showed 1 or more focal lesions. Supervised cluster analysis showed the expression levels of 54 genes that were significantly differentially expressed (greater than 1.4-fold and *P* < 0.0001) between these 2 groups (Figure 1B). *CST6*, coding for cystatin M/E, a soluble inhibitor of cysteine proteases, was the most significantly differentially expressed gene in the analysis (*P* < 0.0001) and was significantly higher in the group with no PET-CT lesions (Figure 1C). Genes associated with cell proliferation were expressed at significantly higher levels in cases with 1 or more PET-CT lesions (Figure 1B). Table 1 shows that, among clinical variables, the absence of PET-CT lesions was associated with a higher incidence of normal albumin levels, a lower incidence of a GEP70 high-risk gene signature (17), higher frequency of gain of chromosome 1q21, and a lower incidence of chromosome 1p deletion and chromosome 5 gain. We found that 67% of the previously defined LB molecular subtype had no PET-CT lesions, while 90% of the proliferation (PR) subtype had 1 or more PET-CT lesions (Table 1 and Supplemental Figure 1A; supplemental material available online with this article; https://doi.org/10.1172/JCI159527DS1). *CST6* was virtually undetectable in PCs isolated from healthy subjects and patients with Waldenstrom’s macroglobulinemia (WM), a BM PC dyscrasia lacking OL (Figure 1C). *CST6* was expressed in a subset of patients with monoclonal gammopathy of undetermined significance (MGUS) and smoldering MM (SMM) (Figure 1C). We have previously shown an inverse relationship between *DKK1* and *CST6* and a strong correlation between *DKK1* and the presence of MRI-defined bone lesions in MM (2). We divided the 512 cases into those in which MM tumor cells expressed either *CST6* or *DKK1* above 5000 relative fluorescence intensity (RFI). *CST6* was more than 5000 in 33 and *DKK1* more than 5000 RFI in 161 (Supplemental Figure 1B). Only one of the 161 cases expressing high *DKK1* also had high *CST6*. None of the 33 cases with high *CST6* had high *DKK1* (Supplemental Figure 1B). These data indicate that elevated *DKK1* and *CST6* define 2 separate subtypes of MM, one with and one without OL bone disease.

Since *CST6* codes for a secreted protein, we developed an ELISA and standard curve for CST6 using recombinant CST6 protein. CST6 was detected in serum isolated from the BM aspirates from which the CD138-purified MM cells were obtained for mRNA microarray studies; serum protein and mRNA levels were correlated (Figure 1D). The mean (±SD) level of CST6 protein in the BM serum/plasma from 75 patients with NDMM for whom gene expression data were also available was 673.0 ± 1076.1 ng/mL. In contrast, the CST6 level was 13.2 ± 19.4 ng/mL in 10 control subjects. These data indicate that the CST6 protein is significantly elevated in MM BM from patients whose tumor cells express high levels of *CST6* mRNA.

### CST6 protein inhibits MM cell–induced bone resorption in vivo.

**Figure 2:**
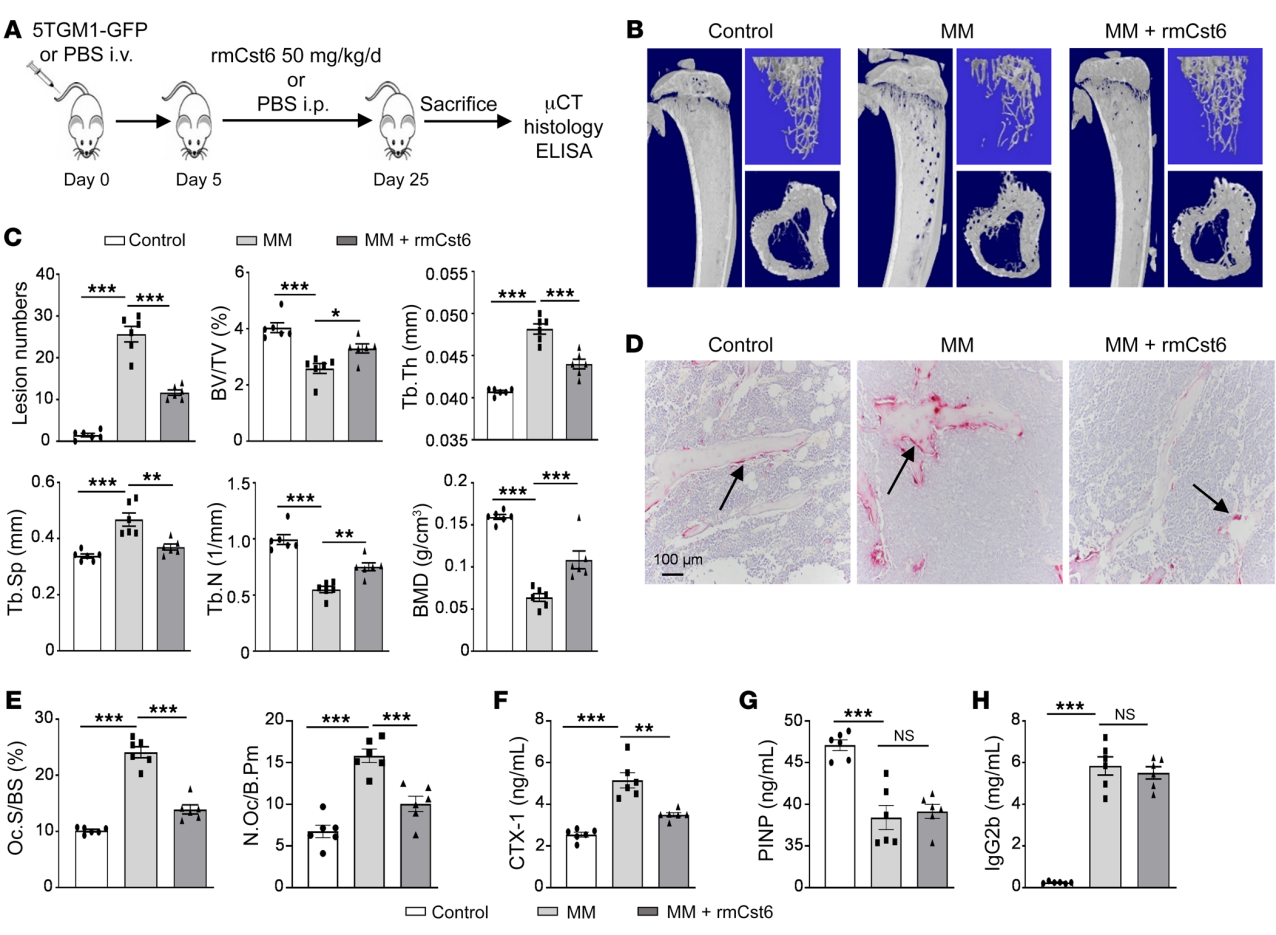
CST6 protein inhibits bone destruction in 5TGM1-C57BL/KaLwRij MM mice. (**A**) Schematic model for the MM mouse study. 5TGM1 murine MM cells were injected into 8-week-old C57BL/KaLwRij female mice via tail vein. rmCst6 protein was administered on day 5 after tumor inoculation. (**B**) Reconstructed μCT images of tibia sagittal sections show bone lytic lesions and trabecular architecture. (**C**) Bar plots present the number of bone lytic lesions on the right medial tibia surface and the trabecular bone parameters: BV/TV, Tb.N, Tb.Th, Tb.Sp, BMD. (**D**) TRAP staining shows osteoclasts (indicated with arrows) in tibiae derived from control C57BL/KaLwRij mice without injection of MM cells and C57BL/KaLwRij mice injected with 5TGM1 MM cells with or without rmCst6 treatment. Scale bar: 100 μm. (**E**) Bar plots represented histomorphometric analyses of TRAP-stained number of osteoclast per bone perimeter (N.Oc/B.Pm) and osteoclast surface per bone surface (Oc.S/BS). (**F** and **G**) Bar plots demonstrated serum levels of the bone-resorption marker CTX-1 and the bone-formation marker PINP detected by ELISA. (**H**) Tumor burden was assessed by measuring serum levels of IgG2b (mg/mL) by ELISA. Data are represented as mean ± SEM (*n* = 6 mice/group) and were analyzed by 1-way ANOVA with Tukey’s multiple comparisons (**C** and **E**–**H**). *P < 0.05; **P < 0.01; ***P < 0.001.

We utilized the 5TGM1-KaLwRij murine MM model (18) to investigate whether recombinant mouse Cst6 protein (rmCst6) could inhibit bone disease in vivo. One million 5TGM1 cells were inoculated into C57BL/KaLwRij mice via the tail vein, and mice were treated with purified rmCst6 (Figure 2A). Intraperitoneal injection of purified rmCst6 protein (50 μg/kg, once per day) significantly decreased OLs in MM-bearing mice (Figure 2, B and C). Micro-computed tomography (μCT) reconstruction of mouse tibiae showed that rmCst6 protein increased trabecular bone volume over total volume (BV/TV), trabecular number (Tb.N), and bone mineral density (BMD) and was accompanied by a decrease in trabecular separation (Tb.Sp) in treated versus control mice (Figure 2, B and C). Histomorphometric analyses demonstrated that rmCst6 administration significantly reduced osteoclast numbers as well as the proportion of bone surface occupied by osteoclasts in MM-bearing mice (Figure 2, D and E). ELISA analyses showed that collagen type 1 (CTX-1), which is a marker of osteoclast activity, was significantly reduced in mice treated with rmCst6 protein (Figure 2F). Serum procollagen type I *N*-propeptide (PINP), a marker of bone formation, did not show any difference between rmCst6-treated and untreated mice (Figure 2G), suggesting that rmCst6 does not alter osteoblast function. Our in vitro study further confirmed that CST6 protein did not influence osteoblast differentiation (Supplemental Figure 2). Serum measurement of the tumor-specific M protein IgG2b after 25 days by ELISA in MM-bearing mice with or without rmCst6 treatment showed no difference between control and rmCst6-treated groups (Figure 2H). We also did not find any evidence that CST6 influenced MM cell proliferation or survival in vitro (Supplemental Figure 3). These data suggest that rmCst6 prevents tumor-induced osteolysis by acting directly on osteoclasts.

### Recombinant human CST6 protein and human MM BM serum with high CST6 protein inhibit osteoclast differentiation and function.

**Figure 3:**
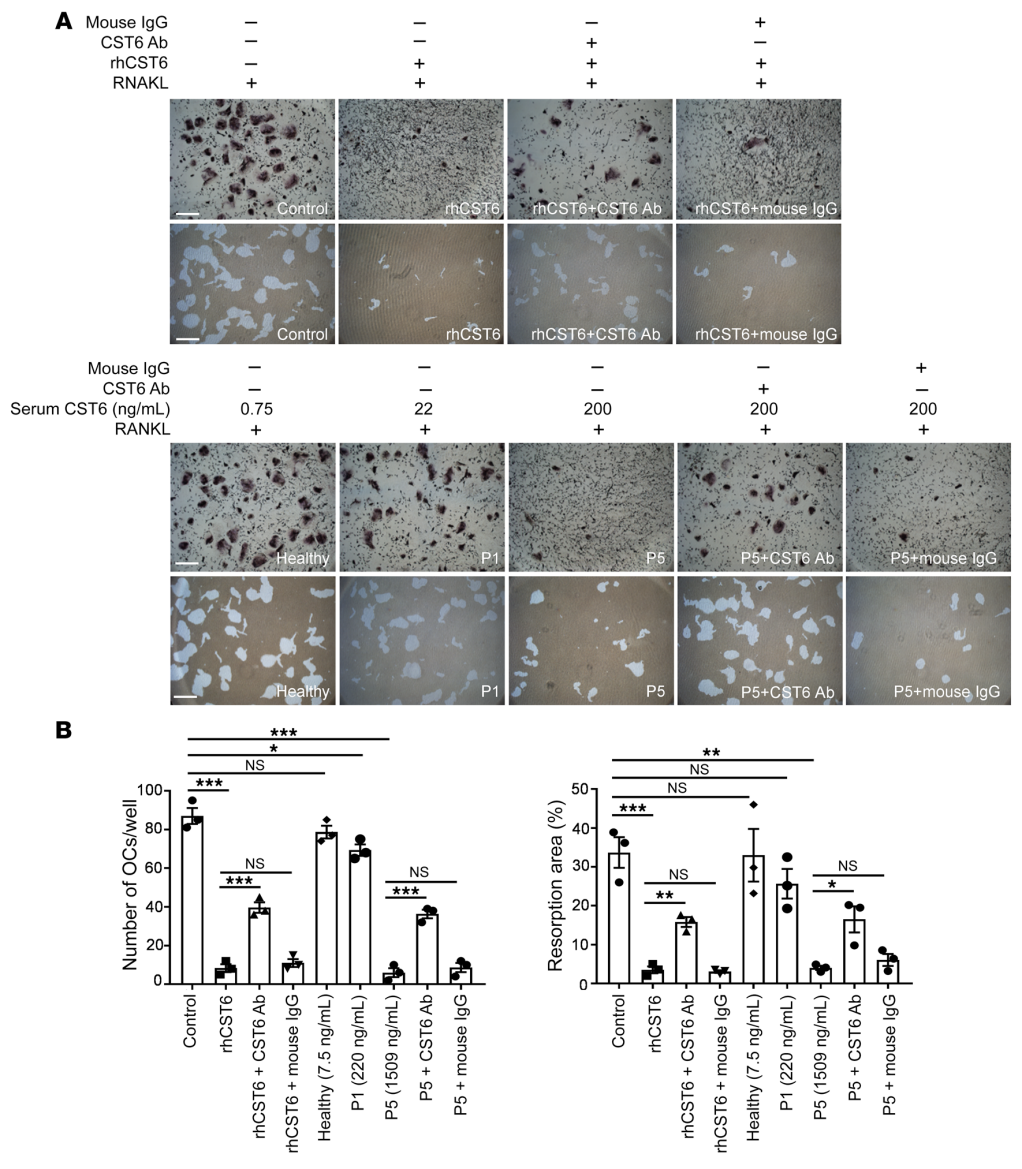
CST6 protein inhibits osteoclast differentiation and function. (**A**) Human OCPs were induced to differentiate into osteoclasts by addition of M-CSF and RANKL. 200 ng/mL rhCST6, 4 μg/mL anti-CST6 antibody, or nonspecific mouse IgG was added to the culture media (upper panels). BM serum from healthy donors and MM patients was added to the culture media with indicated CST6 concentrations (lower panels). On day 7, half of these wells in each group were stained with TRAP solution and the remaining wells were quantified resorption areas. Culture media containing high CST6 protein (final concentration 200 ng/mL) from patient 5 (P5) showed significant decreased TRAP^+^ osteoclasts and bone resorption, while culture media containing low CST6 protein from a healthy donor and patient 1 (P1) with low levels of CST6 did not show these effects. Anti-CST6 antibody or nonspecific mouse IgG (4 μg/mL) was also added to the culture media during human osteoclast differentiation. The CST6 level in each BM serum was determined by ELISA, as described in Supplemental Table 1. Control is represented for RANKL only and was the same as in Supplemental Figure 5 (*n* = 3). Scale bars: 500 μm. (**B**) Bar plots present quantifications of TRAP^+^ osteoclasts and bone-resorption areas. Numbers in parentheses represent CST6 concentrations in patient serum detected by ELISA. Data are represented as mean ± SEM and were analyzed by 2-way ANOVA (**B**). *P < 0.05, **P < 0.01, ***P < 0.001.

As cystatin C, encoded by the *CST3* gene, can decrease osteoclast differentiation (19, 20), we investigated to determine whether CST6 also blocks osteoclast differentiation. Mouse and human BM monocytes were induced to differentiate to osteoclasts by the addition of M-CSF and RANKL with or without different concentrations of rmCst6 and recombinant human CST6 (rhCST6) protein. Tartrate-resistant acid phosphatase (TRAP) staining showed that CST6 significantly suppressed the formation of TRAP-positive multinuclear osteoclasts in a dose-dependent manner (Figure 3, A and B, and Supplemental Figure 4, A and B); this effect was at least partially neutralized by an anti-CST6 antibody, but not by nonspecific IgG (Figure 3, A and B, and Supplemental Figure 4, A and B). Furthermore, CST6 significantly reduced osteoclast resorption areas, as shown using the Corning Osteo Assay, and this reduction was also partially reversed by an anti-CST6 antibody (Figure 3, A and B, and Supplemental Figure 4, C and D). We found that 200 ng/mL rmCst6 protein was sufficient to inhibit osteoclast formation and function (Supplemental Figure 4, A and B). We then evaluated whether BM serum from MM patients with high CST6 expression could prevent osteoclastogenesis. As shown in Figure 3, A and B, and Supplemental Figure 5, A and B, patient-derived BM serum containing 200 ng/mL CST6 protein in the culture media significantly blocked osteoclast differentiation and function, and again, this effect was reversed using an anti-CST6 antibody, but not by nonspecific mouse IgG. In contrast, BM serum from MM patients with low CST6 expression and from healthy donors did not influence osteoclast differentiation and bone resorption (Figure 3, A and B, and Supplemental Figure 5, A and B). These data demonstrate that MM serum with high CST6 can inhibit RANKL-induced osteoclast differentiation.

Cystatin C, encoded by the *CST3* gene, has been shown to prevent bone resorption mainly by inhibiting bone matrix degradation (19, 21) by interfering with the RANKL/RANK signaling pathway in osteoclasts (20) and negatively regulating CTSK activity, which is necessary for bone resorption (22). We found that *CST3* mRNA is highly expressed in PCs derived from healthy donors and MGUS and MM patients (Supplemental Figure 6A). High levels of CST3 were also found in BM serum derived from healthy donors and MM patients (Supplemental Table 1). Using an in vitro assay, CST6 exhibited a 100-fold higher potency in inhibiting osteoclast differentiation and bone resorption compared with CST3 (19, 20) (Supplemental Figure 6, B and C). Our study also showed that an anti-CST6 antibody, but not an anti-CST3 antibody, reversed the effects of high-CST6/high-CST3 MM BM serum in inhibiting osteoclast differentiation and activity (Supplemental Figure 6, D and E), indicating that CST6, but not CST3, plays a critical role in MM osteolytic disease.

### CST6 protein inhibits MM cell–induced bone resorption in an ex vivo model.

**Figure 4:**
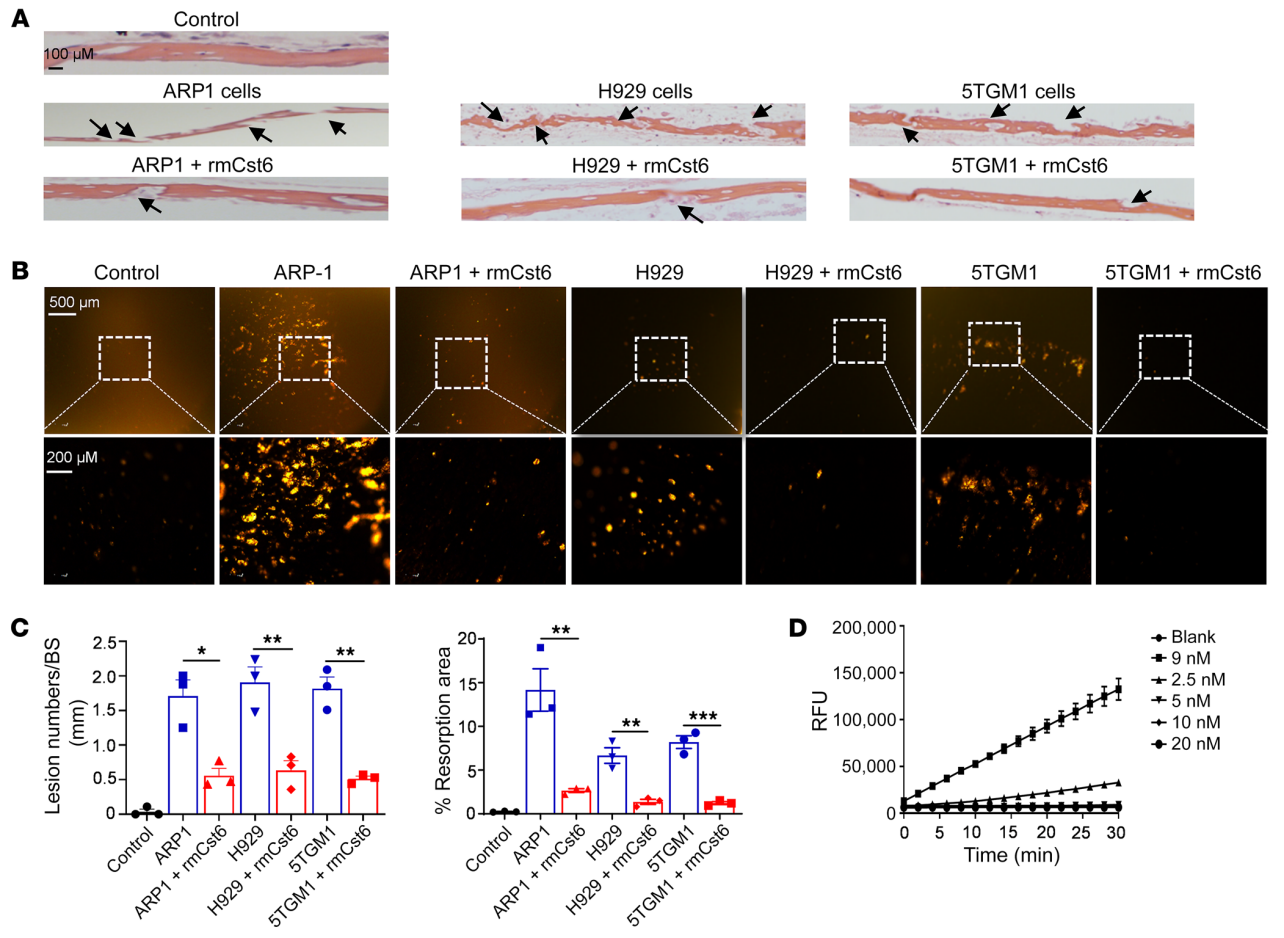
CST6 protein inhibits MM cell–induced bone resorption and CTSK activity. (**A**) Ex vivo organ culture assay was utilized to examine the effect of Cst6 protein on MM-induced bone lesions on calvarias. H&E sections of the parietal bone region showing osteoclastic bone-resorption areas (black arrows) (*n* = 3). Scale bar: 100 μm. (**B**) Silver nitrate staining of calvariae showed the areas with light transparency reflecting bone-resorption areas (*n* = 3). Scale bar: 200 μm. (**C**) Bar plots show the ratio of lytic bone area number to bone surface (left panel) and the percentage of resorption area (right panel) in each group. (**D**) CTSK activity was measured by the Cathepsin K Drug Discovery Kit (Enzo). The *y* axis represents the CTSK activity expressed as relative fluorescence units (RFU); the *x* axis shows time points for treatment with CST6 proteins at different doses (*n* = 3). Data are represented as mean ± SEM. *P < 0.05; **P < 0.01; ***P < 0.001. Statistical analysis was performed using 2-tailed, unpaired Student’s *t* test (**C**).

To further determine the potential role for CST6 in bone biology, we also employed an ex vivo organ culture system (23, 24). MM cells cocultured with mouse calvarial bone cause bone resorption. Human MM cell lines ARP1 and H929 as well as the mouse MM cell line 5TGM1 were cocultured for 10 days with or without rmCst6, with calvarial bone derived from 10-day-old C57BL/6 mice. None of these cell lines express CST6. Both H&E and nitrate silver staining were utilized to evaluate the number of bone-resorption areas (Figure 4, A and B). Quantification of the mean resorption numbers and transparent bone-resorption areas showed that rmCst6 significantly decreased the number of calvarial bone-resorption areas when cocultured with MM cells (Figure 4C). These data demonstrate that CST6 inhibits bone resorption ex vivo.

### CST6 protein inhibits CTSK, an osteoclast-specific protease essential for bone resorption.

Cystatins are inhibitors of lysosomal cysteine proteases, such as cathepsin B, cathepsin L (CTSL), cathepsin V, and LGMN (Supplemental Figure 7, A and C) (25). CTSK is an osteoclast-specific cysteine protease involved in bone catabolism (26). We therefore tested to determine whether CST6 inhibited CTSK activity. An in vitro fluorometric assay showed that CST6 inhibited CTSK in a dose-dependent manner, with a 90% inhibition rate at a dose of 2.5 nM (Figure 4D). These data demonstrate that CST6 blocks the function of CTSK, the cathepsin involved in bone resorption of mature osteoclasts.

### CST6 protein suppresses the bone-resorptive activity of mature osteoclasts.

**Figure 5:**
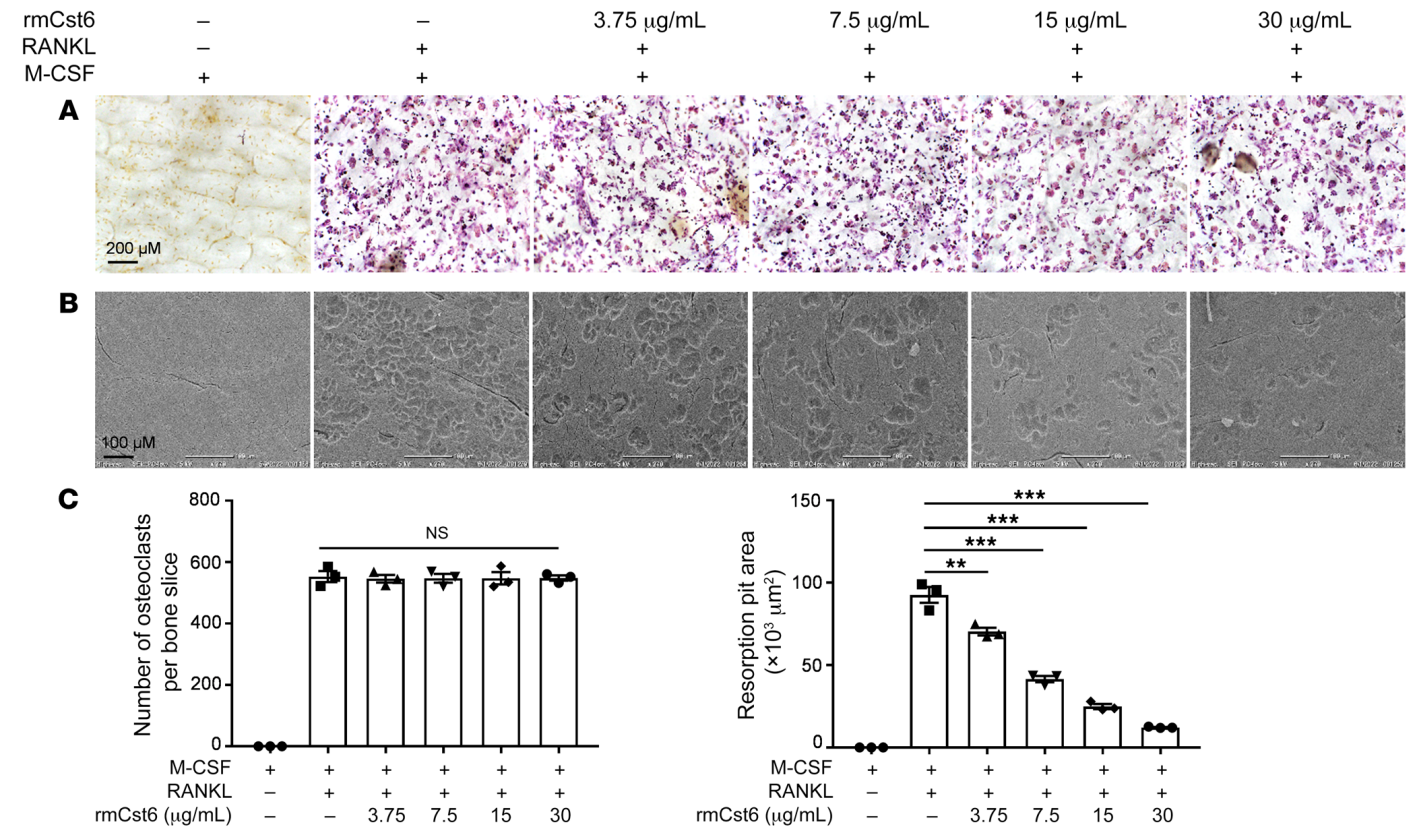
CST6 protein suppresses bone-resorptive activity of mature osteoclasts. (**A**) TRAP staining shows mature osteoclasts on bone slices (*n* = 3). Scale bar: 200 μm. (**B**) After a 3-day culture period, osteoclasts were removed from bone slices, resorption pits were visualized under the SEM, and resorption pit area was quantified (*n* = 3). Scale bar: 100 μm. (**C**) Bar plots show the quantification of TRAP^+^ osteoclasts and the bone-resorption area. Data are represented as mean ± SEM. **P < 0.01; ***P < 0.001. Statistical analysis was performed using 1-way ANOVA with Tukey’s multiple comparisons (**C**).

To determine the effect of CST6 protein on mature osteoclast bone-resorption activity, pre-osteoclast cells were induced to mature osteoclasts with M-CSF and RANKL for 4 days. Equal numbers of mature osteoclasts were seeded on bone slices with or without rmCst6 treatment for 3 days. TRAP staining showed that rmCst6 did not affect the number of mature osteoclasts on bone slices (Figure 5, A–C). Scanning electron microscopy (SEM) showed that 200 ng/mL (13.4 nM) and 500 ng/mL (33.4 nM) rmCst6 did not suppress osteoclast function (Supplemental Figure 8, A–C). However, rmCst6 at a dose of 3.75 μg/mL (0.25 μM) or higher doses significantly suppressed the development of eroded surfaces in a dose-dependent manner, comparable to that seen with the CTSK inhibitor (Figure 5, A–C) (27). These data demonstrate that, at high doses, CST6 blocks the function of mature osteoclasts in vitro.

### CST6 protein attenuates polarization of precursors of osteoclasts and suppresses osteoclast differentiation.

**Figure 6:**
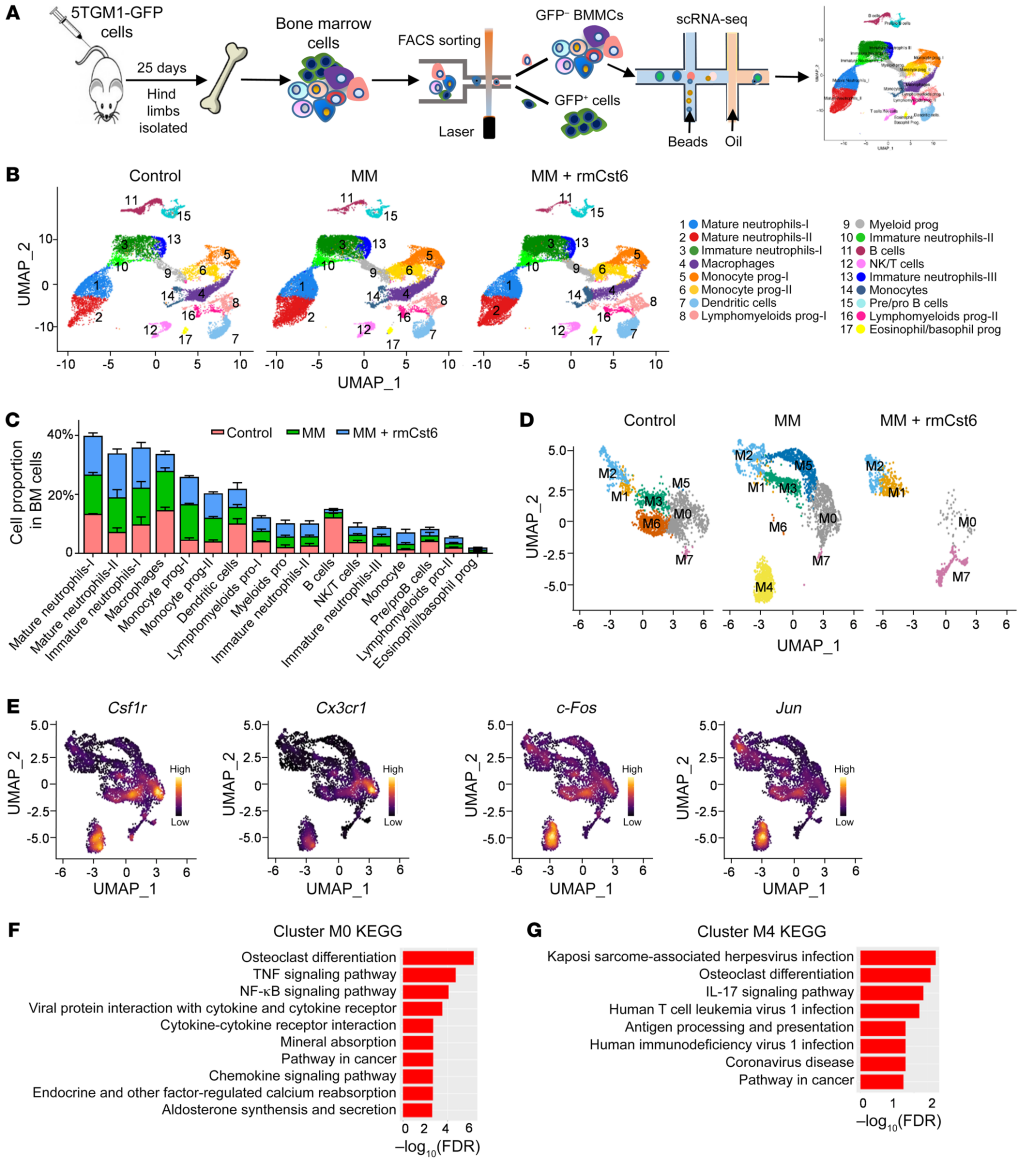
scRNA-Seq reveals that CST6 protein prevents osteoclast differentiation. (**A**) Experimental workflow for scRNA-Seq on BM mononuclear cells. 5TGM1-GFP^+^ MM cells were injected into 8-week-old C57BL/KaLwRij female mice via tail i.v. Hind limbs were extracted, and BM mononuclear cells from individual mice were sorted out by depleting 5TGM1-GFP^+^ MM cells. (**B**) Uniform Manifold Approximation and Projection (UMAP) plot of BM mononuclear cells derived from healthy controls (*n* = 3) and MM-bearing mice treated with PBS (*n* = 3) or rmCst6 protein (*n* = 3). (**C**) Bar plots show the proportion of various cell types in BM mononuclear cells of healthy control and MM-bearing mice treated with PBS or rmCst6 protein. (**D**) UMAP plots of BM macrophages from healthy control (*n* = 3) and MM-bearing mice treated with PBS (*n* = 3) or rmCst6 protein (*n* = 3). (**E**) UMAP plots show expression patterns of marker genes for all clusters collected from 3 groups of mice. (**F**) KEGG pathway analyses show most dysregulated signaling pathways in the cluster M0. (**G**) KEGG pathway analyses show most dysregulated signaling pathways in the cluster M4.

We next performed single-cell RNA-Seq (scRNA-Seq) on BM mononuclear cells from tumor-bearing mice treated with and without CST6. Three groups of mice were included in this study: C57BL/KaLwRij without MM cell injection, C57BL/KaLwRij injected with 5TGM1 cells, and C57BL/KaLwRij injected with 5TGM1 cells and treated with rmCst6 protein for 3 weeks. After removing 5TGM1-GFP^+^ MM cells, BM cells were subjected to scRNA-Seq (Figure 6A). Based on expression profiles, we identified 17 cell populations with differentially expressed cell-specific gene markers (Figure 6B, Supplemental Figure 9B, and Supplemental Table 2). The distribution of these cell types captured by scRNA-Seq was analyzed in control mice and MM mice with or without CST6 protein treatment (Figure 6C). The proportion of BM B cells was dramatically decreased in tumor-bearing mice with or without CST6 treatment (Supplemental Figure 9C), while macrophages were notably decreased in MM mice treated with CST6 protein (Figure 6C and Supplemental Figure 9C).

Because macrophages are precursors of osteoclasts, we further analyzed the population of BM macrophages and identified 8 subclusters using genes variably expressed in macrophages (Figure 6D). Two clusters were dramatically decreased in CST6-treated mice. The cells in one cluster, M0, annotated as early precursors of osteoclasts, exhibited elevated expression of genes of OCPs (*Csf1r* and *Cx3cr1*) (28) (Figure 6E and Supplemental Table 3). The other cluster, M4, mainly appeared in tumor-bearing mice and was dramatically decreased after CST6 protein treatment. Markers of OCPs (*Csf1r*) and early osteoclast differentiation regulators (*c-Fos* and *Jun*) (Supplemental Table 4) are characteristics of M4 (Figure 6E). The osteoclast differentiation pathway was enriched in cluster M0 based on KEGG signaling pathway analysis (Figure 6F). KEGG analysis (https://www.genome.jp/kegg/pathway.html#:~:text=KEGG%20PATHWAY%20is%20a%20collection,Amino%20acid%20Other%20amino%20Glycan) revealed that cluster M4 was enriched for genes in preosteoclast and osteoclast differentiation pathways (Figure 6G) and that cluster M1, M2, M5, and M7 genes were enriched in neurologic disorders and viral infections, but not with osteoclastogenesis (Supplemental Figure 10 and Supplemental Tables 5–8). However, of the M3 gene set, we observed that only 1 gene was differentially expressed when calculating the log-fold change (Supplemental Table 9). This made it impossible to perform a gene-set analysis and interpret gene expression data. With a standard FDR of 0.05, the M6 gene set did not show that signaling pathways were significantly enriched (Supplemental Table 10). Together, these data indicate that CST6 protein suppresses the emergence of OCPs induced by MM cells in the BM.

### CST6 protein selectively suppresses the noncanonical NF-κB signaling pathway in osteoclast differentiation induced by RANKL.

**Figure 7:**
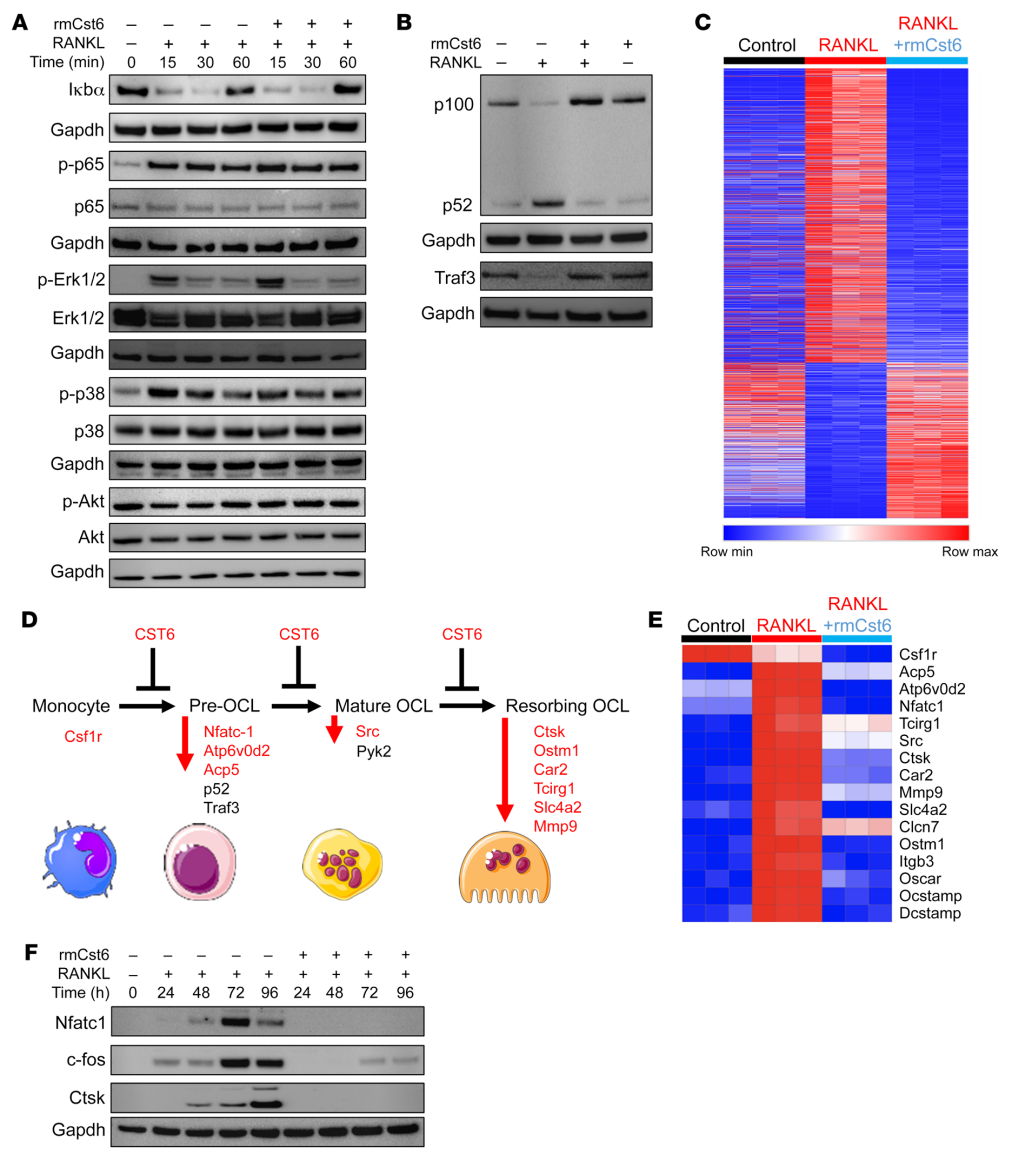
CST6 protein selectively inhibits the noncanonical NF-κB signaling pathway induced by RANKL. (**A**) Mouse OCP (OCP) cells preincubated with 200 ng/mL rmCst6 for 30 minutes were treated with RANKL for indicated times. Western blot shows the expression of Iκbα, p-p65, p65, p-Erk, Erk, p-p38, p38, p-Akt, and Akt (*n* = 3). (**B**) OCPs preincubated with 200 ng/mL rmCst6 for 30 minutes were treated with RANKL for 8 hours. Western blots show the expression of p100/p52 and Traf3 (*n* = 3). (**C**) Heatmap shows 1796 differentially expressed genes (DEGs) between OCPs (control) and OCPs treated with RANKL without or with rmCst6 for 48 hours. (*n* = 3). (**D**) A schematic model for osteoclast differentiation and CST6 functions. Red-labeled genes were significantly downregulated by CST6 protein and are shown in **E**. (**E**) Heatmap shows osteoclast differentiation–associated genes in OCPs (control) and OCPs treated with RANKL without or with rmCst6 for 48 hours. (**F**) Western blot shows the expression of c-fos, Nfatc-1, and CTSK after treatment with rmCst6 at indicated time points in the presence of RANKL (*n* = 3). See complete unedited blots in the supplemental material.

RANKL interacting with its cognate receptor RANK leads to the activation of the NF-κB– and MAPK signaling pathways, which are required for osteoclast formation. To determine whether CST6 protein regulates NF-κB– and MAPK signaling pathways, we preincubated mouse BM macrophages with rmCst6 for 30 minutes and then stimulated these macrophages with RANKL at different time points from 15 minutes to 1 hour. RANKL treatment induced phosphorylation of p65 (p-p65) and induced Iκbα protein degradation after 15 minutes. The presence of rmCst6 protein did not alter p-p65 and Iκbα protein levels, suggesting that Cst6 does not affect the canonical NF-κB pathway (Figure 7A and Supplemental Figure 11). However, rmCst6 inhibited the proteolytic processing of p100 to p52 at 8 hours; rmCST6 also suppressed Traf3 degradation induced by RANKL stimulation (13) (Figure 7B). However, CST6 did not decrease the phosphorylation of Erk1/2, p38, and Akt (Figure 7A and Supplemental Figure 11), suggesting that CST6 does not act on the MAPK signaling pathway. The data suggest that rmCst6 protein prevents RANKL-induced osteoclastogenesis by suppressing the noncanonical NF-κB signaling pathway.

To further determine how CST6 regulates osteoclast differentiation, healthy mouse BM macrophages were isolated and stimulated with RANKL in the presence or absence of rmCst6 protein for 48 hours. RNA-Seq was employed to identify significantly differentially expressed genes between macrophages with and without RANKL induction with or without rmCst6 treatment. As shown in Figure 7C, RNA-Seq analysis identified 1796 genes that were differentially expressed in BM macrophages treated with RANKL plus rmCst6 protein compared with RANKL alone. We found that Cst6 protein inhibited gene expression at different stages of osteoclast differentiation, such as *Csf1r* from monocyte to preosteoclast; *Nfatc1*, *Atp6v0d2*, and *Acp5* (*Trap*) from preosteoclast to mature osteoclast; *Src* from mature osteoclast to resorbing osteoclast; and *Ctsk*, *Ostm1*, *Car2*, *Tcirg1*, *Slc4a2*, and *Mmp9* in resorbing osteoclasts (Figure 7, D and E) (29). We also verified that rmCst6 indeed suppresses protein expression of Nfatc-1, Ctsk, and c-fos in RANKL-treated macrophages (Figure 7F). These data show that CST6 affects the molecular program of osteoclast differentiation.

### CST6 protein suppresses CTSL-induced proteolytic cleavage of p100 and TRAF3.

**Figure 8:**
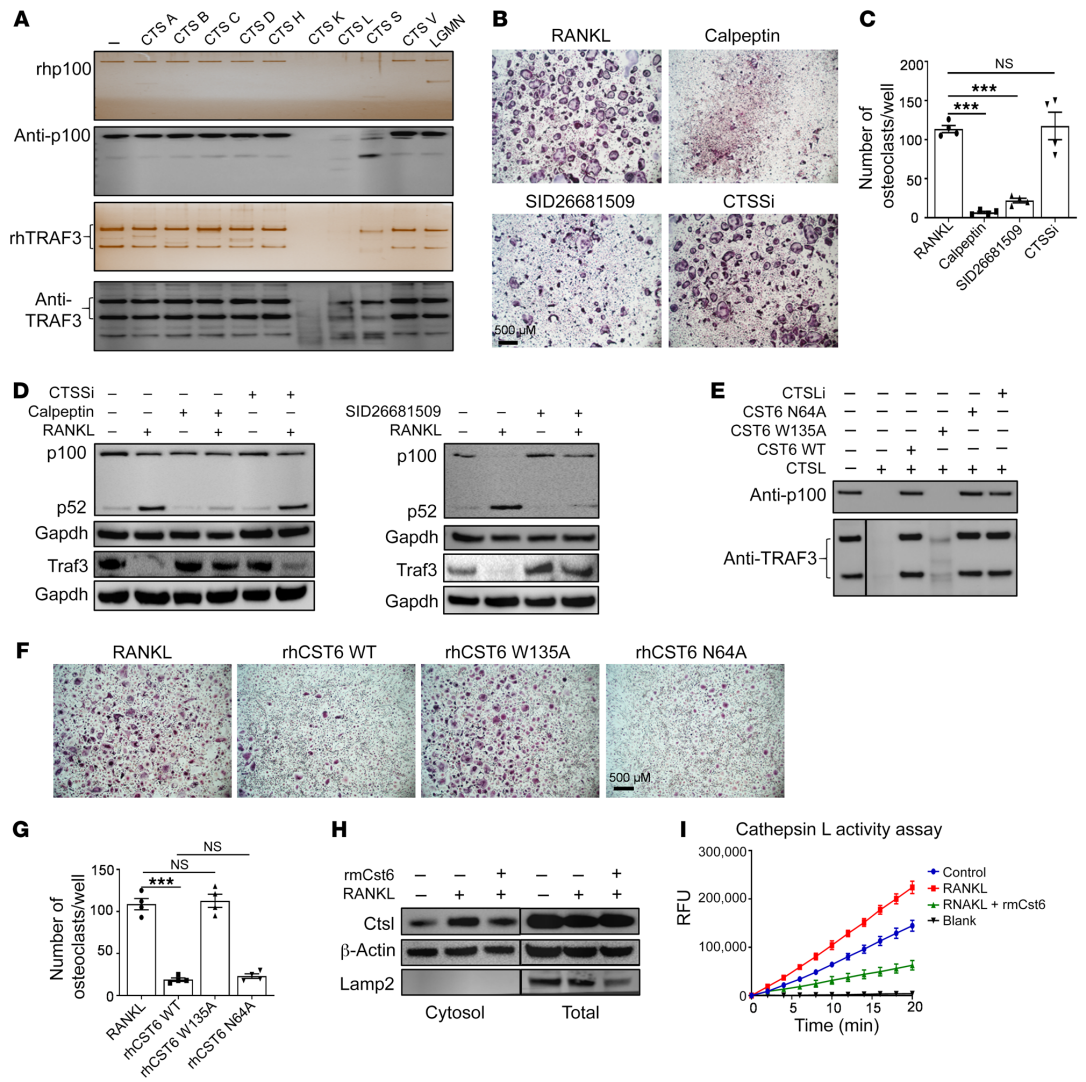
CST6 protein suppresses CTSL-induced proteolytic cleavages of p100 and TRAF3 during osteoclastogenesis. (**A**) Western blots and silver stain show cleaved p100 and TRAF3 proteins (*n* = 3). (**B**) TRAP staining shows osteoclastogenesis suppressed by CTSL inhibitors. Scale bar: 500 μm. (**C**) Bar plots show quantification of TRAP^+^ osteoclasts (*n* = 3). (**D**) OCPs preincubated with 10 μM CTSL inhibitors and CTSS inhibitor for 30 minutes were treated with RANKL for 8 hours. Western blots show the expression of p100, p52, and Traf3 (*n* = 3). (**E**) 20 ng CTSL protein premixed with 20 ng WT or mutant CST6 protein was loaded with recombinant p100 protein and TRAF3 protein in vitro for 30 minutes. Western blots detected the cleaved p100 and TRAF3 proteins (*n* = 3). CTSL inhibitor (CTSLi) was used as a positive control. (**F**) TRAP staining shows osteoclasts treated with 200 ng/mL WT or mutant rhCST6 (*n* = 3). Scale bar: 500 μm. (**G**) Bar plots show quantification of TRAP^+^ osteoclasts. (**H**) Western blots detected increased cytosolic CTSL protein after RANKL induction (*n* = 3). (**I**) CTSL enzymatic activity assay detected CTSL activity from cytosolic protein. The *y* axis represents the CTSL activity expressed as relative fluorescence units; the *x* axis shows time points treated with Cst6 proteins and different controls (*n* = 3). Lanes were run on the same gel, but were noncontiguous (**E** and **H**). Data are represented as mean ± SEM and were analyzed by 1-way ANOVA with Tukey’s multiple comparisons (**C** and **G**). ***P < 0.001. See complete unedited blots in the supplemental material.

Because we found that CST6 stabilizes p100 and TRAF3 proteins, and since CST6 is a natural inhibitor of lysosomal cysteine proteinases and asparaginyl endopeptidases (25), we hypothesized that CST6 protein might suppress lysosomal proteolytic cleavage of p100 and TRAF3 by interacting with its substrates. We performed an in vitro cleavage assay by incubating 10 known substrates of CST6 (cathepsins A, B, C, D, H, K, L, S, and V and LGMN) with p100 or TRAF3 protein, respectively. After 30 minutes of incubation, full-length p100 and TRAF3 proteins were cleaved by CTSL, cathepsin S (CTSS), and CTSK, while other cathepsins and LGMN did not cleave p100 and TRAF3 proteins (Figure 8A). Because CTSK is only expressed in mature osteoclasts, but not in precursors of osteoclast (26), we excluded CTSK being responsible for p100 cleavage in macrophages. To characterize the role of CTSL and CTSS in osteoclastogenesis, mouse BM macrophages were induced to differentiate into cathepsin K–expressing, bone-resorbing osteoclasts by the addition of M-CSF and RANKL with or without 10 μM CTSL and CTSS inhibitors. TRAP staining showed that CTSL inhibitors SID26681509 and calpeptin, but not a CTSS inhibitor, significantly suppressed the formation of TRAP-positive multinuclear osteoclasts (Figure 8, B and C). 10 μM CTSL inhibitors also blocked the p100 and TRAF3 processing induced by RANKL for 8 hours (Figure 8D). CST6 binds and inhibits cathepsins and LGMN through specific binding sites. To verify whether the specific effect of CST6 on p100 and TRAF3 stabilization was through inhibition of CTSL, we cloned and purified human CST6 protein with either a N64A or W135A point mutation, resulting in a selective loss of its suppression function on LGMN and cathepsin activity, respectively (Supplemental Figure 12, A and B). The in vitro cleavage assay of p100 and TRAF3 showed that the W135A mutant CST6 protein did not suppress CTSL activity, while the WT CST6 protein and N64A mutant blocked CTSL cleavage activity (Figure 8E). We also determined the effect of human WT and mutant CST6 protein on human BM osteoclast differentiation; WT and N64A CST6 proteins, but not W135A mutant CST6 protein, blocked osteoclast formation (Figure 8, F and G).

Because p100 and TRAF3 are located in the cytoplasm and ubiquitinated TRAF3 can be degraded in both the cytoplasm and lysosome, we hypothesized that RANKL stimulates the release of CTSL from the lysosome to induce cytoplasmic cleavage of the p100 protein. RAW264.7 cells were treated with RANKL for 48 hours, and cytosolic protein was collected for the CTSL Western blot and activity assays. We found that both the cytosolic CTSL protein levels and activity were increased after RANKL induction (Figure 8, H and I). The data confirm that CTSL protein is released from the lysosome of OCPs following RANKL stimulation.

**Figure 9:**
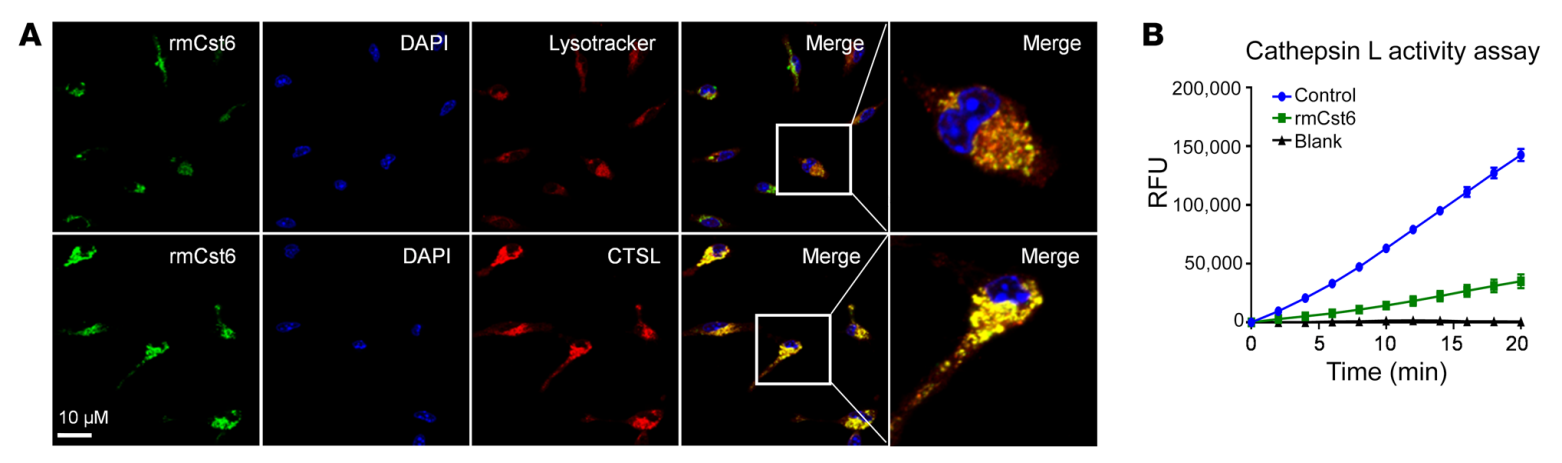
Internalized CST6 protein suppresses CTSL activity in macrophages. (**A**) Macrophages were treated with AF488-labeled rmCst6 for 8 hours and then were costained with CTSL and LysoTracker. Confocal microscope showed the localization of CST6, CTSL, and lysosome (*n* = 3). Scale bar: 10 μm. Original magnification, ×100 (far right panels). (**B**) CTSL enzyme activity assay detected CTSL activity from total protein after treatment with rmCst6. The *y* axis represents the CTSL activity expressed as relative fluorescence units; the *x* axis shows time points of treatment with Cst6 proteins and different controls (*n* = 3).

The secretory CST6 protein can be internalized into lysosomes of melanoma, breast cancer, and lung cancer cells (30). To determine whether MM-derived CST6 protein might be endocytosed into macrophages to suppress CTSL activity and osteoclastogenesis, mouse BM macrophages were isolated and incubated with Alexa Fluor 488–conjugated rmCst6 protein for 8 hours. Fluorescence-labeled rmCst6 protein was detected in the lysosome as well as in the cytoplasm of BM macrophages, as visualized under confocal microscope. To further identify the localization of internalized CST6, BM macrophages were costained with LysoTracker (a unique lysosomal marker; Abcam) and CTSL. We observed a colocalization of CST6 with the LysoTracker and CTSL (Figure 9A). Of note, intracellular CTSL activity of BM macrophages was also inhibited after incubation with rmCst6 protein for 8 hours (Figure 9B). These results demonstrate that CST6 protein can be internalized by macrophages through endocytosis to inhibit CTSL activity and osteoclast differentiation.

## Discussion

Cancer-mediated hyperactivation of genes coding for secreted molecules can have profound effects on the tumor microenvironment. Osteolysis, a hallmark of MM and a severe complication seen in nearly 80% of cases, is strongly linked to the secretion of DKK1 by MM tumor cells (31). In this study, we found that 34% of 512 NDMM patients did not exhibit OL bone disease. To better understand the molecular basis of this difference in tumor behavior, we correlated the presence of PET-CT–defined bone lesions in 512 NDMM patients with global gene expression data derived from CD138-selected BM PCs at diagnosis. These data showed that the absence of bone disease was markedly associated with elevated expression of *CST6*, a cysteine protease inhibitor. Consistent with our previous studies (2), *CST6* was strongly inversely correlated with *DKK1* in the current cohort. *CST6* gene expression levels are correlated with CST6 protein in serum of MM patients. Recombinant CST6 protein or BM serum from MM patients with high CST6 expression inhibits osteoclast differentiation and bone resorption in vitro, and recombinant CST6 suppresses bone loss induced by MM cells as seen in an in vivo MM mouse model. BM serum levels of CST6 in MM can be remarkably high. The highest levels of DKK1 we observed in our previous published studies was 400 ng/mL (2). Most patients with high expression of CST6 were found to have concentrations greater than 400 ng/mL, with the highest being 6913 ng/mL. Based on our extensive clinical experience, patients with MM and high levels of serum CST6 do not exhibit any toxicities that might be linked to elevated CST6, such as skin and hair dysplasias.

Osteolytic bone metastases are common in several solid tumors, including lung and breast cancers, and are a direct cause of morbidity and mortality (32). CST6 is primarily expressed in mammary epithelium, the stratum granulosum of skin epidermis, sweat glands, hair follicles, and nails (25). CST6 has been shown to be downregulated in metastatic breast cancers, and ectopic expression of CST6 prevents bone metastases (25, 33). It is therefore possible that downregulation of CST6 in solid tumors may be a key requirement of the osteolytic metastatic phenotype.

CST6 is a cysteine protease inhibitor that regulates lysosomal cysteine proteases and the asparaginyl endopeptidase LGMN. It is known that CST6 controls the activity of the cysteine proteases cathepsin B (*CTSB*), *CTSL*, cathepsin V (*CTSV*), and transglutaminase-3 (*TGM3*) (34–38). The interaction of CST6 with osteoclast-specific CTSK has, to our knowledge, never been studied. Our in vitro assays demonstrate that CST6 protein inhibits 90% of CTSK activity at 2.5 nM, suggesting that CST6 prevents bone resorption by inhibiting CTSK activity within the ruffled border of the osteoclast.

Whether there is a physiological role for CST6 in normal PC biology is unknown. scRNA-Seq has shown that *CST6* can be expressed in normal PCs (39). The mechanism of *CST6* hyperactivation in MM is unknown. It is not a target of gene-activating, immunoglobulin heavy chain–mediated (IGH-mediated) translocations or copy number changes or point mutations. DNA hypomethylation of the *CST6* promoter may upregulate *CST6* expression in MM cells, but this needs to be studied. MM is characterized by extensive mutations of TRAF3 and other components of the NF-κB signaling cascade (40–42). The LB and MAF/MAFB (MF) molecular subtypes, exhibiting the highest levels of an 11-gene NF-κB gene signature in MM (40), also have the highest expression of *CST6*. Given its potent ability to modulate NF-κB signaling, CST6 may indeed have an NF-κB–related function in MM.

Autocrine CST6 might prevent non–caspase-induced cell death mediated by lysosomal proteases (43). Paracrine CST6 may promote tumor escape from immune surveillance by preventing the protease-dependent presentation of MHC class II molecules on the cell surface (44) or preventing T cell lysosomal protease-mediated cell death (43, 45). We have not observed that CST6 regulates MM cell growth in vitro and in the 5TGM1-C57BL/KalwRij mouse model. Since CST6 blocks RANKL signaling, which is a key pathway for osteoclastogenesis, it may well contribute to the dormancy of tumor cells (46).

Osteoclasts originate from the monocyte-macrophage lineage. RANKL binding to its cognate receptor RANK leads to recruitment of adaptor proteins, such as TNF receptor–associated factors (TRAFs), through which it activates both the canonical and noncanonical NF-κB signaling pathways. The conversion of p105 to p50 is predominantly constitutive, while the processing of p100 to p52 is tightly regulated by a TRAF3/NIK/IKKα axis (12). Ubiquitinated TRAF3 has been reported to be degraded in both the lysosome and proteasome after RANKL stimulation (13). However, it is not clear which protease or proteases degrade TRAF3 protein in the lysosome. In our study, we found that CTSL activated by RANKL directly cleaves not only TRAF3 but also p100, a major suppressor of noncanonical NF-κB signaling. CTSL-knockout mice show increased resistance to osteoporosis following ovariectomy (47). We further demonstrate that RANKL-induced CTSL leakage from the lysosome results in direct cleavage and degradation of p100 protein, initiating osteoclastogenesis. In agreement with our observation, Li et al. have reported that RANKL stimulates the release of cathepsin B from the lysosome to the cytoplasm (33). Although cathepsin B could not directly cleave p100 and TRAF3 proteins, CST6 can suppress cathepsin B cleavage of SPHK1, which prevents osteoclastogenesis (33).

Recently, single-cell transcriptome analysis has been employed to define the comprehensive cellular makeup of tumor and tumor microenvironmental cells (48–51). We used this technology to dissect the BM microenvironment alterations influenced by CST6 protein, especially in macrophages, precursors of osteoclasts. We observed that MM cells induced polarization of precursors of osteoclasts and this effect was blocked by CST6 protein. In combination with RNA-Seq data, we further determined that CST6 protein inhibited gene expression at different stages of osteoclast differentiation, such as pre-, mature, and resorbing osteoclasts.

Not all MM patients with osteolytic bone disease express high levels of *DKK1*. Likewise, elevated *CST6* is not seen in all cases lacking osteolytic bone lesions. This could reflect the stage of disease at the time of diagnosis or heterogeneity in tumor cell gene expression in a systemic disease and/or could point to the existence of other mechanisms underlying the development or suppression of OL in MM. Nevertheless, the experimentally validated results of our correlative studies integrating both imaging and genomics in MM strongly suggest that CST6 represents a potent regulator of bone biology and potentially a new biological anti–bone-resorptive agent for the treatment of disease associated with osteoclast-mediated bone loss.

## Methods

### Patients.

We analyzed 512 NDMM patients who had Affymetrix U133Plus2.0 microarray and PET-CT data at diagnosis. Table 1 shows the characteristics of the patients with MM.

### PC isolation and GEP.

GEP and sample preparation were performed as previously described (52). The results of GEP were deposited in the NCBI’s Gene Expression Omnibus database (GEO GSE2658).

### Bone imaging.

Fluorodeoxyglucose-PET/CT (FDG-PET/CT) was performed as previously described (53). All imaging studies were interpreted by a team of experienced radiologists and nuclear medicine physicians well versed in myeloma diagnostics who had no prior knowledge of the gene expression data.

### 5TGM1/KaLwRij mouse model.

Six- to eight-week-old female C57BL/KaLwRij mice were injected with either 100 μL PBS or 1 × 10^6^ 5TGM1-GFP cells i.v. via the tail vein and randomized into 3 groups (*n* = 6/group). After 5 days of injection of tumor cells, mice were treated with either PBS or rmCst6 (50 μg/kg) via i.p. injection every day. On day 25 after tumor cell inoculation, when most mice had started to develop paraplegia, the experiment was terminated and mice were sacrificed. Blood samples were collected every week.

### μCT.

Mouse tibiae were dissected and fixed in 10% neutral-buffered formalin for 2 days. μCT of mouse tibia was performed by using SkyScan1272 scanner (Bruke). Scans were acquired at 60 kV and 166 μA, Al 0.5 mm filter,10 μM pixel size. After scanning, tibia images were reconstructed using the Skyscan NRecon program, version 2.0, with a beam-hardening correction of 40. Trabecular and cortical bone microarchitecture were analyzed using the Skyscan CT Analyzer program, version 2.0. OLs on the curved medial tibial surface that completely penetrated the cortical bone and were greater than 100 μm in diameter were counted (54).

### Bone histomorphometry.

Following μCT, the same tibiae were decalcified in 5% EDTA solution (pH 7.0) for 7 days at room temperature and embedded in paraffin. Bone sections (5 μm thickness) were stained with H&E and TRAP using a Leukocyte Acid Phosphatase Kit (Sigma-Aldrich). Histomorphometric analyses were performed using OsteoMeasure software, version 7.0 (OsteoMetrics), with a Zeiss Axioskop2 microscope (Carl Zeiss).

Further details are available in Supplemental Methods and Supplemental Table 11.

### Data availability.

Bulk RNA-Seq data and scRNA-Seq data have been deposited in the GEO database (GSE191187 and GSE191258, respectively).

### Statistics.

Results are presented as mean ± SEM, as indicated in figure legends. Statistical analysis was done using GraphPad Prism, version 7.0. All other comparisons were analyzed by unpaired, 2-sided, independent Student’s *t* test, unless otherwise described in the figure legends. One-way ANOVA or 2-way ANOVA was used to determine the statistically significant difference for multiple group comparisons. A *P* value of less than 0.05 was considered to indicate statistical significance (55).

### Study approval.

The animal studies were performed according to guidelines of the Institutional Animal Care and Use Committee (IACUC) at the University of Arkansas for Medical Sciences under an approved protocol (IACUC 3991). The Institutional Review Board of the University of Arkansas for Medical Sciences approved these research studies, and all subjects provided informed consent approving use of their samples for research purposes. Deidentified primary samples were obtained from myeloma patients during clinic visits at the University of Arkansas for Medical Sciences. Signed Institutional Review Board–approved informed consent forms are kept on record at the University of Arkansas for Medical Sciences Tissue Biorepository and Procurement Service (TBAPS) under approved protocols IRB 262254 and 260887. Peripheral blood from healthy donors was collected using UARK protocol 2009-88 under IRB 5455.

## Author contributions

DG and JRC performed the experiments, collected and analyzed the data, generated the figures, and wrote and edited the manuscript; IN analyzed the scRNA-Seq data. CA analyzed the RNA-Seq data. JPS, FS, YC, CL, HX, BP, TKG, CS, ST, MZ, FC, BB, and FVR reviewed the data and provided clinical data and relevant interpretation. HH guided biochemical experiments for functional studies of CST6 and cathepsins. GT analyzed and interpreted data and contributed to the writing of the manuscript. JDS and FZ conceptually developed this project, designed and supervised this study, collected and analyzed data, and wrote and edited the manuscript. All authors discussed the results and commented on the manuscript.

## Supplementary Material

Supplemental data

Supplemental table 1

Supplemental table 2

Supplemental table 3

Supplemental table 4

Supplemental table 5

Supplemental table 6

Supplemental table 7

Supplemental table 8

Supplemental table 9

Supplemental table 10

## Figures and Tables

**Table 1:**
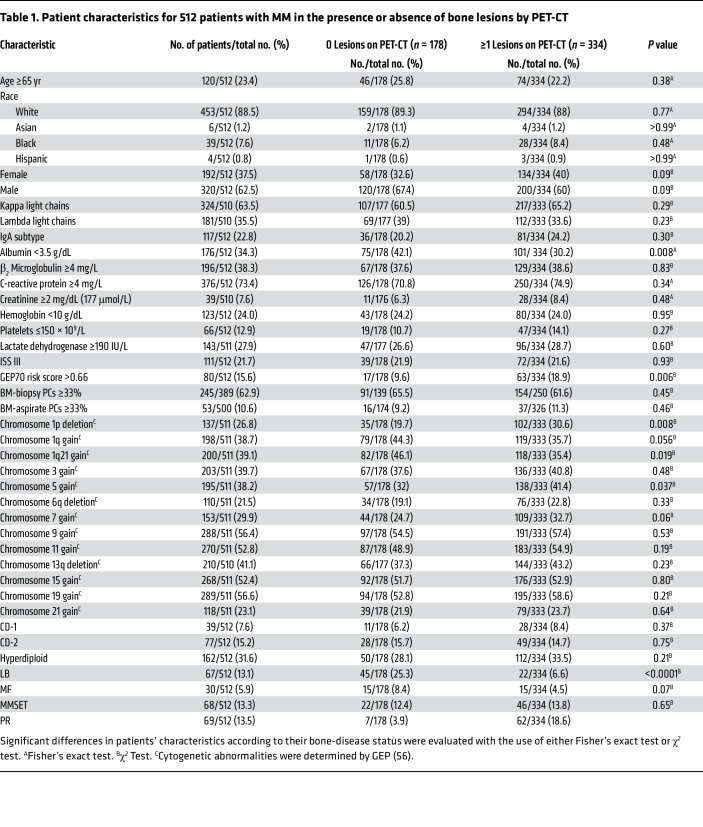
Patient characteristics for 512 patients with MM in the presence or absence of bone lesions by PET-CT

## Version 1 07/26/2022

In-Press Preview

## Version 2 09/15/2022

Electronic publication

## Version 3 09/21/2022

Update to acknowledgments
