# Supplementary material for: CST6 suppresses osteolytic bone disease in multiple myeloma by blocking osteoclast differentiation
Source: J Clin Invest. 2022 Sep 15;132(18):e159527. doi: 10.1172/JCI159527 (PMC9479617; doi:10.1172/JCI159527)
Supplement: Supplemental data [file jci-132-159527-s011.pdf]

Supplemental Methods

Plasma cell isolation and gene-expression profiling

Gene expression profiling (GEP) and sample preparation were performed as previously described (1). Briefly, Plasma cell (PC) isolation from mononuclear cell fractions was performed by immunomagnetic bead selection with monoclonal mouse anti–human CD138 antibody with the use of the AutoMACS automated separation system (Miltenyi-Biotec). PC purity of > 95% homogeneity was confirmed by 2-color flow cytometry with the use of CD138+/CD45− criteria (Becton Dickinson), immunocytochemistry for cytoplasmic light-chain immunoglobulin, and morphology by Wright-Giemsa staining. RNA was extracted with the QIAGEN RNeasy kit. cDNA was prepared and biotinylated with the Affymetrix GeneChip HT 3′ IVT Express Kit. Samples were hybridized to an Affymetrix U133Plus2.0 microarray according to the manufacturer's recommendations and then read on a GeneChip Scanner 3000 (Affymetrix). The results of gene-expression profiling were deposited in Gene Expression Omnibus (http://www.ncbi.nlm.nih.gov/geo/. opens in new tab) under the accession number GSE2658.

CST6 sandwich ELISA

Nunc™ MaxiSorp™ ELISA Plates (Biolegend) were coated with 50 µl of a monoclonal CST6 antibody (R&D, catalog # MAB1286) at a concentration of 2 µg/ml in ELISA coating buffer overnight at 4 °C. The plates were washed and blocked with 1% bovine serum albumin (100 µl/well) at room temperature for 1 h. Plates were washed prior to addition of recombinant CST6 protein (R&D, catalog # 1286-PI-010) for establishment of a standard curve (0-100 ng/ml in ELISA dilution buffer), conditional media of MM cell lines and patient serum/plasma (1:100) samples to each well of the plates and incubated at 4 °C overnight. Plates were washed before incubation with biotinylated polyclonal anti-CST6 antibody (50 µl/well, 0.2 µg/ml in PBS, pH 7.2) (R&D, catalog # BAF1286) at room temperature for 2 h. Plates were then washed prior to incubating each well with 50 µl of a 1:10,000 dilution of streptavidin-horseradish peroxidase (ThermoFisher, catalog # N100) at room temperature for 1 h. Color development was achieved with the substrate (R&D, catalog # DY999) according to manufacturer’s instructions and the reaction was stopped by treatment of the plates with 2M sulfuric acid (50 µl/well, 0.5 mol/l). The absorbance values were measured at 450 nm.

CST3 sandwich ELISA

Nunc™ MaxiSorp™ ELISA Plates (Biolegend) were coated with 50 µl of a polyclonal CST3 antibody (R&D, catalog # AF1196) at a concentration of 2 µg/ml in ELISA coating buffer overnight at 4 °C. The plates were washed and blocked with 1% bovine serum albumin (100 µl/well) at room temperature for 1 h. Plates were washed prior to addition of recombinant CST3 protein (R&D, catalog # 1196-PI-010) for establishment of a standard curve (0-100 ng/ml in ELISA dilution buffer), conditional media of MM cell lines and patient serum/plasma (1:1000) samples to each well of the plates and incubated at 4 °C overnight. Plates were washed before incubation with biotinylated polyclonal anti-CST3 antibody (50 µl/well, 0.2 µg/ml in PBS, pH 7.2) (R&D, catalog # BAF1196) at room temperature for 2 h. Plates were then washed prior to incubating each well with 50 µl of a 1:10,000 dilution of streptavidin-horseradish peroxidase (ThermoFisher, catalog # N100) at room temperature for 1 h. Color development was achieved with the substrate (R&D, catalog # DY999) according to manufacturer's instructions and the reaction was stopped by treatment of the plates with 2 M sulfuric acid (50 µl/well, 0.5 mol/l). The absorbance values were measured at 450 nm.

Cell cultures

All cells were maintained at 37°C in a 5% CO2 incubator. The myeloma cell line ARP-1 was generated in University of Arkansas for Medical Sciences, murine myeloma cell line 5TGM1 was provided by Claire M. Edwards. H929 and 293T cell lines were purchased from American Type Culture Collection (ATCC). ARP-1, 5TGM1 and H929 were cultured in RPMI1640 medium with 10% fetal bovine serum (FBS) in the presence of penicillin (100 U/mL) and streptomycin (100 mg/mL). RAW264.7 and 293T cell lines were maintained in Dulbecco's modified Eagle's medium (DMEM) with 10% FBS in the presence of penicillin (100 U/mL) and streptomycin (100 mg/mL).

Animal models

C57BL/KaLwRij mice used in this study were obtained from Harlan Laboratory Inc. (Harlan Mice, Netherlands) and 6-8 weeks female C57BL/KaLwRij mice were employed for 5TGM1-GFP cells injection or scRNA-seq experiments. All animals were housed under specific pathogen free conditions on a 12 h light/dark cycle with stable temperature (22 °C) and humidity (60%) at the University of Arkansas for Medical

Sciences. All animal procedures adhered to a protocol approved by the local Institutional Animal Care and Use Committee at University of Arkansas for Medical Sciences.

Evaluation of tumor burden and bone turnover markers

5TGM1/KaLwRij mice were bled every week to harvest serum for detecting IgG2b by ELISA according to the manufacturer's instructions (Bethyl Laboratories). The serum levels of CTX-1 and PINP were examined by ELISA using a CTX-1 ELISA kit (MyBioSource, catalog # MBS722404) and PINP ELISA kit (MyBioSource, catalog # MBS4501850) according to the manufacturer's instructions.

RNA isolation and RNA sequencing (RNA-seq)

BM monocytes obtained from C57BL6 mice were differentiated into osteoclasts in the presence of M-CSF and RANKL for 48 h. Cells were processed for RNA extraction using the Quick-DNA/RNA FFPE Miniprep Kit with on-column DNase digestion for the RNA preps (catalog # D7003; Zymo Research, Irvine CA, USA). RNA was assessed for mass concentration using the Qubit RNA Broad Range Assay Kit (catalog # Q10211; Invitrogen, Waltham MA, USA) with the Qubit 4 fluorometer (catalog # Q33238; Invitrogen, Waltham MA, USA). RNA integrity was assessed with a Standard Sensitivity RNA Analysis Kit (catalog # DNF-471-0500; Agilent, Santa Clara CA, USA) on the Fragment Analyzer System (catalog # M5310AA; Agilent, Santa Clara CA, USA). Sequencing libraries were prepared using TruSeq Stranded mRNA Library Prep (catalog # 20020595; Illumina, San Diego CA, USA) with the IDT for Illumina Unique Dual indices (catalog # 20022371; Illumina, San Diego CA, USA). Libraries were assessed for mass concentration using the Qubit 1X dsDNA HS Assay Kit (catalog # Q33231; Invitrogen, Waltham MA, USA) with the Qubit 4 fluorometer (catalog # Q33238; Invitrogen, Waltham MA, USA). Library fragment size was assessed with the High Sensitivity NGS Fragment Analysis Kit (catalog # DNF-474-0500; Agilent, Santa Clara CA, USA) on the Fragment Analyzer System (catalog # M5310AA; Agilent, Santa Clara CA, USA). Libraries were functionally validated with the KAPA Universal Library Quantification Kit (catalog # 07960140001; Roche, Basel CH). Sequencing was performed to generate paired-end (2X100) reads with a NovaSeq 6000 200-cycle SP flow cell (Illumina, San Diego CA, USA).

Single cell RNA sequencing (scRNA-seq)

At 25 days post-tumor cell inoculation in the 5TGM1/KaLwRij mice, BM mononuclear cells were isolated by Ficoll-Paque PLUS. BM microenvironmental cells were sorted out using fluorescence-activated cell sorting (FACS) by depleting 5TGM1-GFP$^+$ MM cells. Sorted GFP negative cells with a purity greater than 95% and viability higher than 95% were used for 10 X genomics scRNA-seq. Cells were resuspended in Mg-free and Ca-free PBS with 0.04% BSA and counted using a light microscope under 10X magnification with Trypan Blue and diluted to the desired concentrations to target approximately 10,000 cells according to protocol recommendations. Single-cell emulsions were generated with the Chromium Controller (10X Genomics, Pleasanton, CA, USA) using the Chromium Next GEM Chip G (Part Number 1000120, 10X Genomics) with the Chromium NextGEM Single Cell 3' v3.1 kit (Part Number 1000121, 10X Genomics) following the standard protocol. Libraries were assessed for mass concentration using the Qubit 1X dsDNA HS Assay Kit (catalog # Q33231; Invitrogen, Waltham MA, USA) using the Qubit 4 fluorometer (catalog # Q33238; Invitrogen, Waltham MA, USA). Library fragment size was assessed with the High Sensitivity NGS Fragment Analysis Kit (catalog # DNF-474-0500; Agilent, Santa Clara CA, USA) on the Fragment Analyzer System (catalog # M5310AA; Agilent, Santa Clara CA, USA). Libraries were functionally validated with the KAPA Universal Library Quantification Kit (catalog # 07960140001; Roche, Basel CH). Initial low-pass "surveillance" sequencing was performed on an Illumina NovaSeq SP 100-cycle flow cell and data was assessed using the Cell Ranger Count output.

Bioinformatic analysis of scRNA-seq

The raw sequencing data (fastq files) were preprocessed using CellRanger software (10X Genomics) version 6 with reference genome of *Mus musculus* version mm10 to demultiplex for cell and transcript and generate count table. The count table was loaded into R through Seurat version 4 package (2) for further analysis. The cells that have number of gene lesser than 500, greater 5,000, and more than 10% of unique molecular identifiers stemming from mitochondrial genes were discarded out from the analysis. For individual sample, Principal component analysis (PCA) was then performed on significantly variable genes of the remained high-quality cells. The results of individual sample were used for data integration across samples using reciprocal PCA method to minimize technical differences between samples. Then the

nitration results were used as input for clustering using Louvain algorithm with multilevel refinement (3) and Uniform Manifold Approximation and Projection for Dimension Reduction (UMAP). The gene specific markers of each cluster were identified using the FindMarkersAll function with MAST method (4) test statistics. The expression patterns of selected gene markers across cell cluster were plotted as a heat map to assess the quality of cell clustering result. After quality control and filtering, we obtained high quality single-cell transcriptomes from ~8000 cells per mouse (**supplemental Figure 9A**). These cell populations and markers included mature neutrophils-I (*S100a8, Ly6g*), mature neutrophils-II (*Rethlg, MMP9*), immature neutrophils-I (*Chil3, Camp*), macrophages (*Adgre1, Mafb*), monocyte prog-I (*F13a1, Itga1*), monocyte prog-II (*Prtn3*, *Hsp90ab1*), dendritic cells (*Siglech, Bst2, Tcf4*), lymphomyeloids prog-I (*Irf8, Flt3*), myeloid prog (*Mpo*, *Cd63*), immature neutrophils-II (*Cenbf, Camp*), B cells (*Ighm, Cd79b*), NK/T cells (*Cd3e, Gzmb*), immature neutrophils-III (*Fcnb, Camp*), monocytes (*ly6c2, ccl2*), pre/pro B cells (*Pax-5, Vpreb3*), lymphomyeloids prog-II (*Atp2b4, Hlf*) and eosinophil/basophil prog (*Gata2, Cpa3*) (**supplemental Figure 9B and Table S2**).

To visualize some of gene markers simultaneously in kernel joint density estimatiscon, the Nebulosa package was used (5). Based on the kernel joint density of Adgr1 and Fcgr3, we sub-select cells that have high value of the kernel joint density for subclsutering analysis to study the cellular heterogeneity of Macrophage cells. Gene-set enrichment analysis of marker genes was performed on Gene Ontology annotation using piano package (6).

Cathepsin K activity assay

The ability of CST6 (R&D, catalog # 1286-PI-010) to inhibit cathepsin K protease activity was assessed using the cathepsin K drug discovery kit (Enzo, catalog # BML-AK430-0001). Fluorimetric assays were done in triplicate in 96-well microtitre plates using cathepsin K, the fluorogenic synthetic substrate Z-Phe-Arg-AMC in the presence of the cathepsin K assay buffer. Recombinant CST6 was added to assays at various concentrations and the resultant fluorescence was measured using a Biotek Synergy Plate Reader (BioTec) with excitation at 380 nm and emission at 460 nm wavelengths. The initial rates from the cathepsin K progress curves in the presence of predetermined concentrations of recombinant CST6 protein were subjected to Graphpad software to allow determination of the inhibition rate.

Cathepsin B activity assay

The ability of CST6 (R&D, catalog # 1286-PI-010) to inhibit cathepsin B protease activity was assessed using the cathepsin B inhibitor screen kit (Abcam, catalog # ab185438). Recombinant CST6 was added to assays at various concentrations and the resultant fluorescence was measured using a Biotek Synergy Plate Reader (BioTec) with excitation at 400 nm and emission at 505 nm wavelengths in a kinetic mode for 30 min at 37 °C.

Cathepsin L activity assay

The ability of CST6 (R&D, catalog # 1286-PI-010) to inhibit cathepsin L protease activity was assessed using the cathepsin L inhibitor assay kit (Abcam, catalog # ab197012). Recombinant CST6 was added to assays at various concentrations and the resultant fluorescence was measured using a Biotek Synergy Plate Reader (BioTec) with excitation at 400 nm and emission at 505 nm wavelengths in a kinetic mode for 30 min at 37 °C.

Cathepsin V activity assay

Cathepsin V activity was measured by cleavage of the Z-LR-AMC fluorogenic peptide substrate (R&D Systems, catalog # ES008). Dilute the rhCathepsin V (R&D Systems, catalog # 1080-CY-010) to 1 µg/ml with assay buffer (25 mM Sodium Acetate, 0.1 M NaCl, 5 mM DTT, pH 5.5). 50 µL was loaded into a black well plate and treated with 10 µL rCST6 protein at various concentration. After incubating for 30 min at 37 °C, 50 µl substrate solution (final concentration to 40 µM) was added into each well. The fluorescence was monitored at 380 nm excitation/460 nm emission in kinetic mode for 30 min with the Biotek Synergy Plate Reader (BioTec).

Preparation of osteoclasts and osteoclast resorption assays

Human osteoclast precursor cells (Lonza, catalog # 2T-110) were re-suspended to a final concentration of 50,000 cells/mL, and 100 µL with 5,000 cells were plated per well in a 96 tissue culture plate and 96 well Corning® Osteo Assay Surface plate (Corning, catalog # 3988) in α-MEM medium include 10% FBS, 25 ng/ml M-CSF (Biolegend, catalog # 574804) and 50 ng/ml RANKL (R&D, catalog # 390-TN-010/CF) in the

absence or presence of CST6 for 7 days. Half-media changes were carried out every 3 days. The cells were then fixed in formalin and stained for TRAP using a TRAP staining kit. $TRAP^+$ cells containing 3 or more nuclei were counted as osteoclasts. To analyze the surface for pit formation, the media was aspirated from the wells on day 7, and 100 μL of 10% bleach solution was added. Cells were incubated with the bleach solution for 5 min at room temperature. The wells were washed twice with distilled water and allowed to dry at room temperature for 3 to 5 h. Resorption pits were photographed and analyzed by image J software (National Institutes of Health). For patient BM serum effects on osteoclast differentiation, the serum CST6 concentration in P4-P6 was normalized to 2000 ng/ml, then 10 μl serum was added to the 100 μl culture media (final concentration 200 ng/ml). For the healthy donor and MM patients P1-P3, 10 μl serum from each sample was added to the 100 μl culture media, and the final concentration was 0.75, 22, 29.6, and 8.4 ng/ml, respectively.

For mouse cell studies, primary mouse BM cells were collected from 6-8 week C57BL/6 mice, 4 x 10^4 cells were seeded into 96-well plates with α-MEM containing 10% FBS and 10 ng/ml M-CSF (PeproTech, catalog # 315-02) for 3 days to recruit macrophage and then induce osteoclast differentiation plus 10 ng/ml RANKL (R&D, catalog # 462-TEC-010/CF) with or without CST6 for 3-5 days. Half-media changes were carried out every 2 days. The cells were then evaluated for TRAP staining and pit resorptions as described above.

Preparation of osteoclasts from MM patients bone marrow cells

MM patients BM mononuclear cells (BMMCs) were positively selected using anti-human CD14 Microbeads (Miltenyi Biotec, catalog # 130-050-201). 4 x 10^4 cell were seeded into 96-well plates in α-MEM medium including 10% FBS, 25 ng/ml M-CSF (Biolegend, catalog # 574804) and 50 ng/ml RANKL (R&D, catalog # 390-TN-010/CF) for 14 days. Half-media changes were carried out every 2-3 days. The cells were then evaluated for TRAP staining and pit resorptions as described above.

Resorption pit assay

Primary mouse BM cells were collected from 6-8-week C57BL/6 mice, 4 x 10^4 cells were seeded into 96-well plates with α-MEM containing 10% FBS and 10 ng/ml M-CSF (PeproTech, catalog # 315-02) for 3 days

to recruit macrophage and then induce osteoclast differentiation plus 10 ng/ml RANKL (R&D, catalog # 462-TEC-010/CF) for 3-5 days. Mature osteoclasts were lifted by trypsin and 4 x 10^4 cells were seeded on the bone slices (Immunodiagnosticsystems, catalog # DT-1BON1000-96) with or without CST6 treatment for 3 days. Then, TRAP staining was performed, and the $TRAP^+$ multinucleated cells (nuclei ≥ 3) were quantified. After $TRAP^+$ OCL counts were determined, bone slices were incubated in ddH2O with 1% bleach and sonicated until cells were removed from the bone surface. The eroded area on the bone slices was visualized by scanning electron microscopy (JCM-6000; JEOL, Tokyo, Japan). Resorption pits were photographed and analyzed by image J software (National Institutes of Health).

Osteogenic differentiation assay

Osteoblast precursors MC3T3-E1 were seeded at 15,000 cells/cm^2 in 12-well or 24-well plates. At 80% confluence, the media were changed to osteogenic induction medium (α-MEM without ascorbic acid, supplemented with 10% FBS, 1% penicillin-streptomycin, 50 µg/ml L-Ascorbic Acid (Sigma-Aldrich, catalog # A5960) and 5 mM β-Glycerophosphate (Sigma-Aldrich, catalog # 9422). The media were changed every 2–3 days during the culture period. On day 14, the activity of alkaline phosphatase (ALP) was evaluated. Cells were washed with PBS and fixed with 4% paraformaldehyde for 20 min at room temperature, and then stained with 1-step NBT-BCIP substrate solution (Thermo Scientific, catalog # 34042) for 20 minutes. On day 21, cells were fixed and stained with 2% Alizarin Red S (Sigma-Aldrich, catalog # A5533) at pH 4.2 to evaluate cell matrix mineralization.

Ex vivo organ culture assay

Calvariae from 10-day old neonatal C57BL/6 mice were dissected as described (7, 8). Half calvarial pieces were co-cultured with 2 x 10^5 MM cells in α-MEM/RPMI1640 (Invitrogen) 50/50 medium supplemented with 1% Penicillin/Streptomycin (Invitrogen) for 10 days in six-well plates, the upper well was loaded with a calvaria and the lower well was seeded with myeloma cells*.* The media were changed every 3 days. At the end of the experiment, half of the calvariae were fixed in 10% formalin for 24 h, decalcified for 48 h in 10% EDTA pH7.2, embedded in paraffin, sectioned and stained with H&E. The whole length of the slides was captured using the Olympus BX-61 microscope. The quantitative representation of the ex vivo organ culture

assay (EVOCA) was calculated bone lesion numbers and normalized with the whole bone length. Other half of the calvariae were fixed in 10% formalin overnight and counter stained with 2% silver nitrate (Sigma-Aldrich, catalog # S8157) for 1 h. The mineral loss and bone resorption areas were clearly apparent under the microscope where resorption regions were transparent to light. The transparent areas were quantified and calculated under 10x magnification.

CST6 expression and purification

Human and mouse CST6 cDNAs cloned into pcDNA3.1(+)-C-6His were purchased from GenScript (Piscataway, NJ). The constructs were transfected into HEK293T cells via Lipofectamine2000 (ThermoFisher, catalog # 11668500). Conditioned media was collected at 48 h and 72 h after transfection. The pH of the medium was adjusted to pH 7.5-8.0 with 0.05 M NaOH, then loaded into the HisTrap HP column (Cytiva, catalog # 17524801) using a peristaltic pump at 4 °C. The His tag proteins were washed with 50 ml of 50 mM Na-Phosphate, 300 mM NaCl, 10% glycerol, 5 mM Imidazole pH 7.5, and eluted with 50 mL 0-100% to 50 mM Na-Phosphate, 300 mM NaCl, 10% glycerol, 300 mM Imidazole pH 7.5 using the NGC column Chromatography System (Bio-Rad, Hercules, CA). After concentration by ultrafiltration, 5 ml samples were loaded onto a Superdex 75 100/300 GL column (Cytiva, catalog # 29148721) pre-equilibrated with 50 mM Na-Phosphate pH 7.5, 150 mM NaCl, at a flowrate of 0.75 ml/min. The protein purity was determined by silver stain according to the Pierce Silver Stain Kit protocol (ThermoFisher, catalog # 24612). The concentration of the purified protein was determined at 280 nm by nanodrop 2000 (Thermo Scientific). The purified protein was tested for functionality prior to use in *in-vivo* tests.

The CST6 mutants N64A and W135A were constructed with the QuickChange site-directed mutagenesis kit (ThermoFisher, catalog # F541) as previously described (9). The protein purification of CST6 mutants is the same as described above. The protein activity was tested and confirmed functionally prior to use.

In vitro cleavage reactions

100 ng recombinant human p100 protein (OriGene, catalog # TP313313) was incubated with vehicle or 20 ng recombinant human Cathepsin A, B, C, D, H, K, L, S, V and LGMN (R&D Systems) at 35°C for 30 min

in a buffer containing 20 mM Tris-HCl, pH 7.2, 100 mM NaCl, 3 mM MgCl2, 0.1% Triton X-100, 5% Glycerol, 1 mM DTT. 100 ng recombinant human TRAF3 protein (OriGene, catalog # TP318682) was incubated with vehicle or 20 ng recombinant human Cathepsin A, B, C, D, H, K, L, S, V and LGMN (R&D Systems) at 35°C for 30 min in a buffer containing 20 mM Tris-HCl, pH 5.0, 100 mM NaCl, 3 mM MgCl2, 0.1% Triton X-100, 5% Glycerol, 1 mM DTT. The reaction was stopped by immediately adding the SDS loading buffer and boiling. The cleavage of p100 and TRAF3 was then monitored by Western Blotting.

Cytosol protein isolation

20 x 10^6 RAW264.7 cells were harvested and washed with ice-cold PBS. Washed pellets were homogenized with a dounce homogenizer (30-50 strokes) in 2 ml detergent -free isotonic homogenize buffer (20 mM Hepes-KOH pH 7.5, 1 mM EDTA, 0.25 M sucrose, 1 mM DTT) with Protease inhibitor (ThermoFisher, catalog # 32965), and checked for achieving 90% cell breakage under the phase contrast microscope. The cell debris was spin down at 15,000 g for 15 min, and the supernatant was used as cytosol fraction. The pellets (including nuclei, membrane, mitochondria and lysosome) were lysed on ice for 30 min in a lysis buffer containing 150 mM NaCl, 10 mM EDTA, 10 mM Tris pH 7.4, and 1% Triton X-100 supplemented with Protease inhibitor, followed by centrifugation at 13,500 rpm for 10 min at 4°C.

Cytosol cathepsin L activity assay

20 x 10^6 RAW264.7 cells were harvested and washed with ice-cold PBS. Washed pellets were homogenized with Dounce homogenizer (30-50 strokes) in 2 ml detergent -free isotonic homogenize buffer (20 mM Hepes-KOH pH 7.5, 1 mM EDTA, 0.25 M sucrose, 1 mM DTT) and checked for achieving 90% cell breakage under the phase contrast microscope. The cell debris was spin down at 15,000g for 15 min, and the supernatant was used as cytosol fraction. The protein concentration was quantified by BCA Protein Assay Kit (ThermoFisher). Cathepsin L activity was measured by cleavage of the Z-LR-AMC fluorogenic peptide substrate (R&D Systems). 10 µl Cell lysate (20 µg) was added to black 96-well microplates (ThermoFisher). After the addition of 40 µl buffer (100 mM Sodium Citrate, 1 mM EDTA, 0.1% CHAPS, pH 5.8) and 50 µl substrate solution (final concentration to 40 µM). The fluorescence was monitored at 380 nm excitation/460nm emission in kinetic mode for 20min with Biotek Synergy Plate Reader (BioTec).

Western blot

Cells were treated with CST6 protein at indicated concentration and durations. Cells were lysed in 150 mM NaCl, 10 mM EDTA, 10 mM Tris pH 7.4, and 1% Triton X-100 supplemented with Protease inhibitor (ThermoFisher). Protein lysates were incubated on ice for 30 min and centrifuged at 13,500 rpm for 4℃ for 10 min. Proteins were separated with NuPAGE 4% to 12% Bis-Tris Gel (Invitrogen) at 200 V, then transferred to a nitrocellulose membrane for 1 h at 400 mA at 4°C. The membrane was blocked for 60 min with 5% milk at room temperature. Primary antibodies IKBα (Cell Signaling Technology, catalog # 9242S,1:1000), p65 (Cell Signaling Technology, catalog # 8242S,1:1000), p-p65-Ser536 (Cell Signaling Technology, catalog # 3033S,1:1000), ERK-1/2 (Cell Signaling Technology, catalog # 4695S,1:1000), p-ERK1/2- Thr202/Tyr204 (Cell Signaling Technology, catalog # 4370S,1:1000), p38 (Cell Signaling Technology, catalog # 9212S,1:1000), p-p38-Thr180/Tyr182 (Cell Signaling Technology, catalog # 9211S,1:1000), p100/52 (Cell Signaling Technology, catalog # 4882S,1:1000), AKT (Cell Signaling Technology, catalog # 9272S,1:1000), p-AKT-T450 (Cell Signaling Technology, catalog # 9267S,1:1000), c-Fos (Cell Signaling Technology, catalog # 2250S,1:1000), NFATc-1 (Sant Cruz Biotechnology, catalog # sc7294,1:500), CathepsinK (Sant Cruz Biotechnology, catalog # sc48353,1:500), CathepsinL (Sant Cruz Biotechnology, catalog # sc390367,1:500), Lamp2 (Sant Cruz Biotechnology, catalog # sc20004,1:500), TRAF3 (Sant Cruz Biotechnology, catalog # sc6933,1:200), GAPDH(Sant Cruz Biotechnology, catalog # sc365062,1:1000), were incubated overnight. Secondary antibodies were incubated for 1 h at a concentration of 1: 10,000. For exposure, ECL Prime Wstern Blotting Detection Reagent (Cytiva, catalog # RPN2236) was used. Imaging was done with a Bio-Rad ChemiDoc XRS+ with Image Lab Software.

LysoTracker staining

5000 mouse primary macrophages coated on the cover slip were incubated with Alexa Fluor-488 conjugated mouse CST6 protein for 8 h. The macrophages were co-stained with Lysotracker (Abcam, catalog # ab112137) for 30 min at 37°C. The fluorescent signal was then visualized using a Zeiss LSM 880 confocal microscope.

Immunofluorescence staining

5000 primary mouse macrophages coated on the cover slip were incubated with Alexa Fluor-488 conjugated mouse CST6 protein (Alexa Fluor 488 Conjugation Kit, catalog # ab236553) for 8 h. The macrophages were fixed in 4% paraformaldehyde and permeabilized with 0.25% Triton X-100 in 1 x PBS. After blocking with 1% BSA in 0.25% PBST, the cells were stained with antibody against CTSL (R&D, catalog # AF1515-SP) at 4°C overnight. Alexa 594-labeled conjugated anti-goat secondary antibody (Abcam, catalog # ab150132) was used for detection. The fluorescent signal was then visualized using a Zeiss LSM 880 confocal microscope.

References:

1. Shaughnessy JD, Jr, Qu P, Usmani S, Heuck CJ, Zhang Q, Zhou Y, Tian E, Hanamura I, van Rhee F, Anaissie E, et al. Pharmacogenomics of bortezomib test-dosing identifies hyperexpression of proteasome genes, especially PSMD4, as novel high-risk feature in myeloma treated with Total Therapy 3. *Blood.* 2011;118(13):3512-24.
2. Stuart T, Butler A, Hoffman P, Hafemeister C, Papalexi E, Mauck WM, Hao Y, Stoeckius M, Smibert P, and Satija R. Comprehensive Integration of Single-Cell Data. *Cell.* 2019;177(7):1888-902.e21.
3. Blondel VD, Guillaume J-L, Lambiotte R, and Lefebvre E. Fast unfolding of communities in large networks. *Journal of Statistical Mechanics: Theory and Experiment.* 2008;2008(10):P10008.
4. Finak G, McDavid A, Yajima M, Deng J, Gersuk V, Shalek AK, Slichter CK, Miller HW, McElrath MJ, Prlic M, et al. MAST: a flexible statistical framework for assessing transcriptional changes and characterizing heterogeneity in single-cell RNA sequencing data. *Genome Biology.* 2015;16(1):278.
5. Alquicira-Hernandez J, and Powell JE. Nebulosa recovers single-cell gene expression signals by kernel density estimation. *Bioinformatics.* 2021;37(16):2485-7.
6. Väremo L, Nielsen J, and Nookaew I. Enriching the gene set analysis of genome-wide data by incorporating directionality of gene expression and combining statistical hypotheses and methods. *Nucleic Acids Res.* 2013;41(8):4378-91.

7. Mohammad KS, Chirgwin JM, and Guise TA. Assessing new bone formation in neonatal calvarial organ cultures. *Methods Mol Biol.* 2008;455(37-50.

8. Xu L, Mohammad KS, Wu H, Crean C, Poteat B, Cheng Y, Cardoso AA, Machal C, Hanenberg H, Abonour R, et al. Cell Adhesion Molecule CD166 Drives Malignant Progression and Osteolytic Disease in Multiple Myeloma. *Cancer Research.* 2016;76(23):6901-10.

9. Cheng T, Hitomi K, van Vlijmen-Willems IM, de Jongh GJ, Yamamoto K, Nishi K, Watts C, Reinheckel T, Schalkwijk J, and Zeeuwen PL. Cystatin M/E is a high affinity inhibitor of cathepsin V and cathepsin L by a reactive site that is distinct from the legumain-binding site. A novel clue for the role of cystatin M/E in epidermal cornification. *The Journal of biological chemistry.* 2006;281(23):15893-9.

Figure S1

A

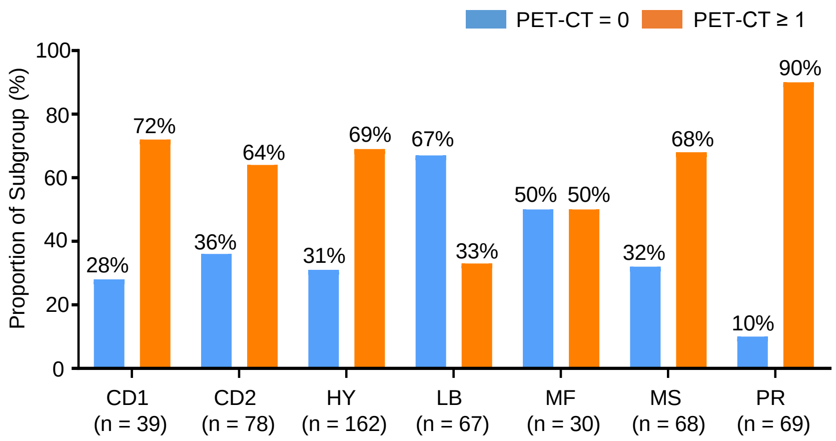

B

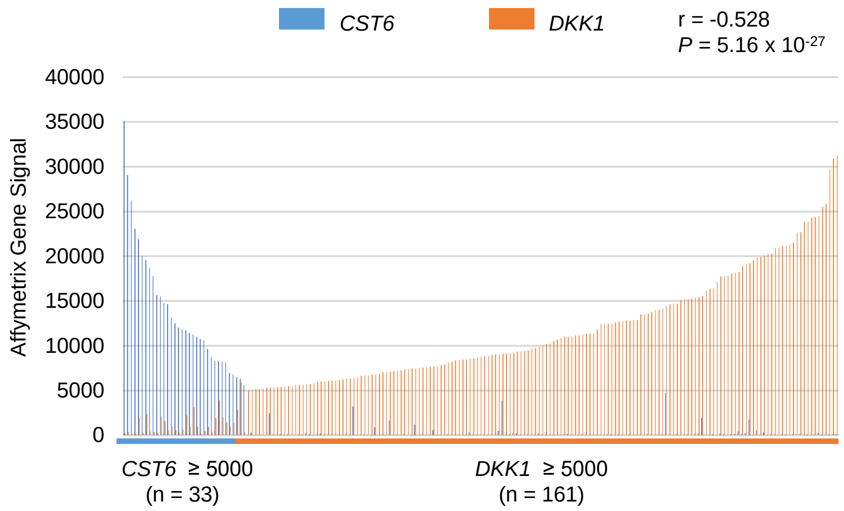

Figure S1. The expression correlation of *DKK1* and *CST6* in primary human MM samples. Related to Figure 1. (**A**) A bar-view presents the proportion of patients with no PET-CT lesions or with 1 or more PET-CT lesions for each MM subtype. (**B**) A bar-view shows *DKK1* and *CST6* expression. The expression of *CST6* and *DKK1* in each sample is indicated by the height of the bar. A negative correlation was showed between *CST6* and *DKK1* signal with a threshold at 5000. *P* value was obtained by Pearson's correlation and linear regression analysis.

Figure S2

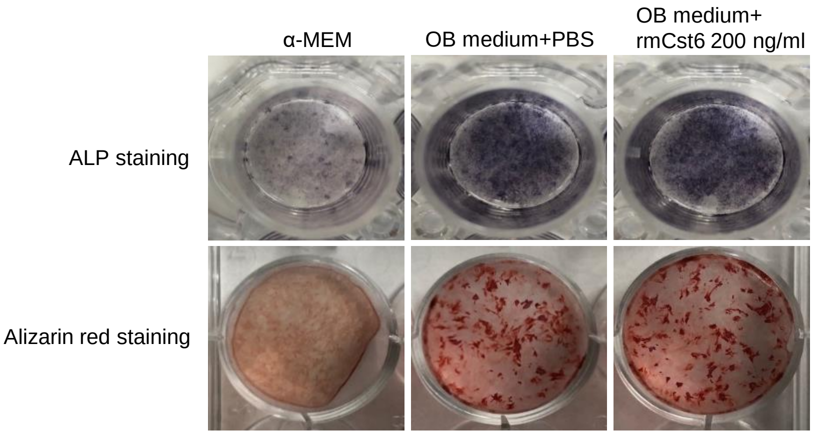

Figure S2. CST6 does not influence osteoblast differentiation of MC3T3-E1 cells. Related to Figure 2. Alkaline phosphatase staining and Alizarin red staining were performed on MC3T3-E1 cells treated with PBS or rmCst6 at the dose of 200 ng/ml for 14 days and 21 days (n = 3).

Figure S3

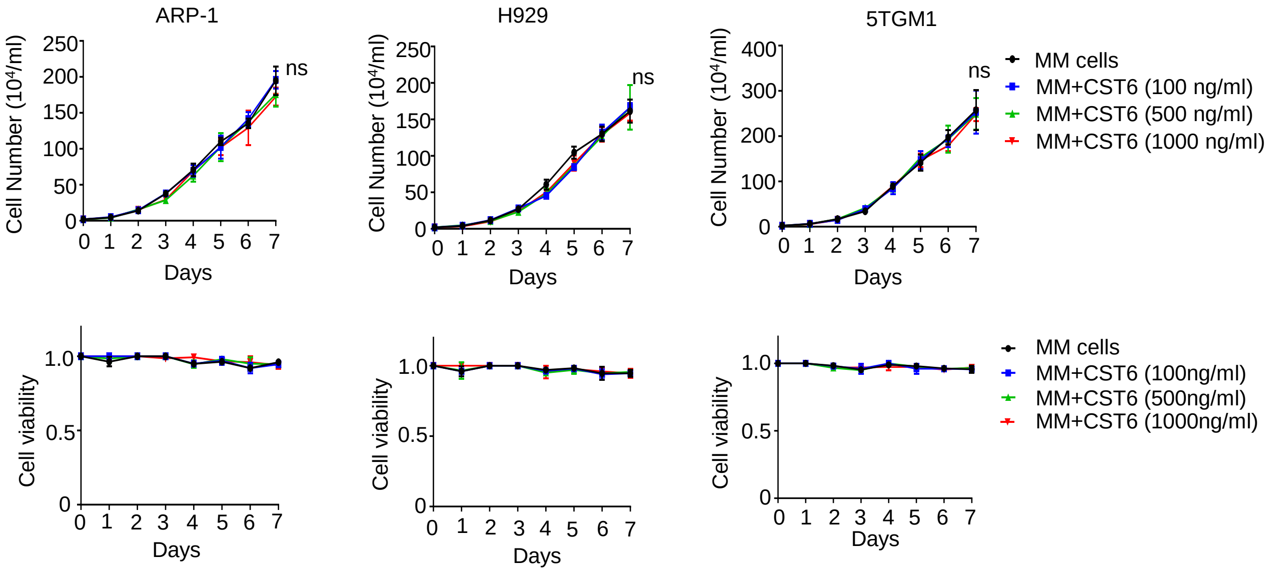

Figure S3. CST6 does not alter MM cell viability or proliferation. Related to Figure 2.
ARP-1, H929, and 5TGM1 MM cell lines were cultured with different doses of CST6 protein for 7 days, cell growth and viability were assessed by trypan blue (n = 3). Growth and survival curves show that recombinant CST6 doesn't influence the cell proliferation and viability. Results shown as mean ± SEM. Statistical analysis was performed using 2-way ANOVA, ns, not significant.

Figure S4

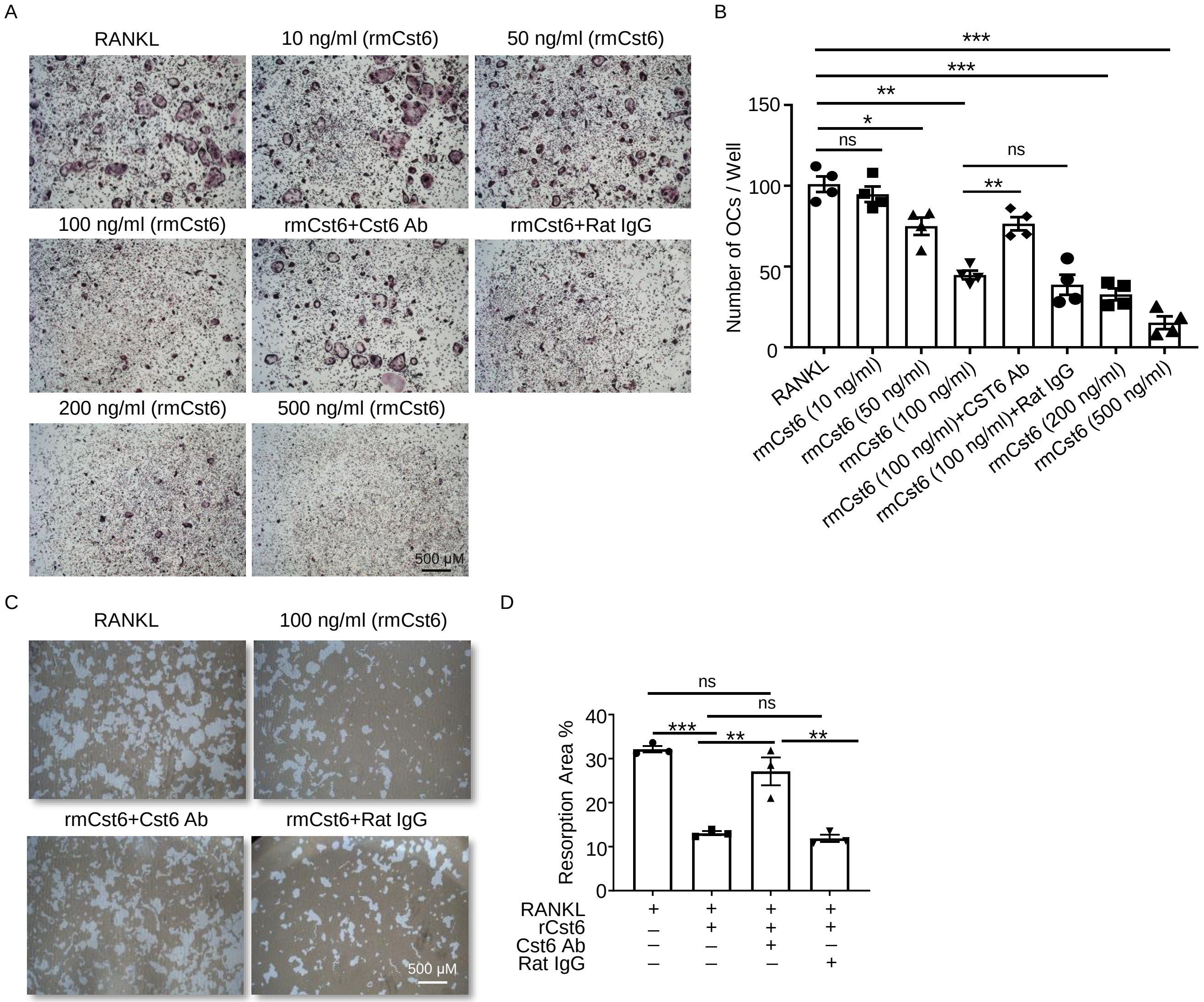

Figure S4. CST6 protein inhibits RANKL-induced osteoclast differentiation and bone resorption. Related to Figure 3.
(**A**) Mouse BM monocytes were seeded into 96-well plates at a density of 4 x 10^4 cells/well and cultured with different concentrations of recombinant mouse Cst6 protein as indicated in the figure, +/- anti-Cst6 antibody, or +/- non-specific rat IgG for 4 days, TRAP staining shows osteoclasts containing multiple nuclei (n = 3). (**B**) Bar-plots showed the quantification of $TRAP^+$ osteoclasts. (**C**) 4 x 10^4 cells/well mouse BM monocytes were seeded into a Corning® Osteo Assay plate, bone resorption areas in the plate were measured in the presence of RANKL, +/- rmCst6, +/- anti-Cst6 antibody, or +/- non-specific rat IgG for 4 days (n = 3). (**D**) Bar-plots showed quantification of the bone resorption areas. Scale Bar = 500 μm. Data were presented as mean ± SEM and analyzed by two-way ANOVA (**B** and **D**). **P* < 0.05; ***P* < 0.01; ****P* < 0.001; ns, not significant.

Figure S5

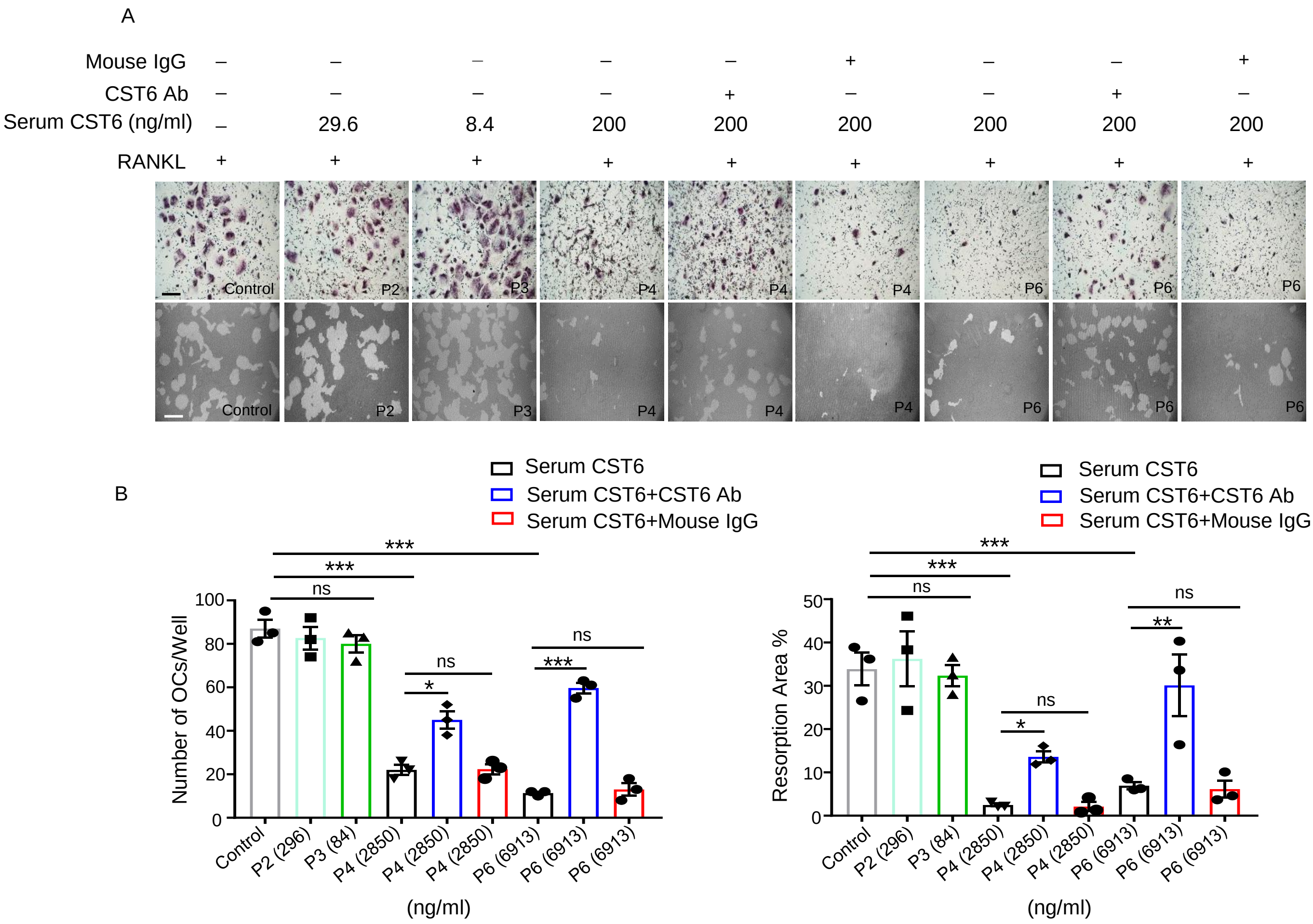

Figure S5. CST6 protein inhibits osteoclast differentiation and function. Related to Figure 3.

(**A**) Human osteoclast precursors were differentiated into osteoclasts with M-CSF and RANKL. BM serum from MM patients were added into the culture media with indicated CST6 concentrations. Culture media containing high CST6 protein (final concentration 200 ng/ml) from Patient 4 (P4) and Patient 6 (P6) showed significantly inhibition of osteoclast differentiation and bone resorption, while culture media containing low CST6 protein from Patient 2 (P2) and Patient 3 (P3) with low levels of CST6 did not show this effect. 4 μg/ml of anti-CST6 antibody or non-specific mouse IgG were also added to the culture media during human osteoclast differentiation. On day 7, half of the wells in each group were stained with TRAP solution and the remaining wells were quantified resorption areas. Scale Bar = 500 μm. The CST6 level in each BM serum was determined by ELISA as described in Table S1. Control represented for RANKL only and shared the same control with Figure 3A. (**B**) Bar-plots presented the results of TRAP^{+} osteoclasts and the bone resorption area quantification (n = 3). Data were presented as mean ± SEM and analyzed by two-way ANOVA (**B**). The statistical analysis accounts for the total number of comparisons made in both Figure 3 and Figure S5A. *P < 0.05, **P < 0.01, ***P < 0.001; ns, not significant.

Figure S6

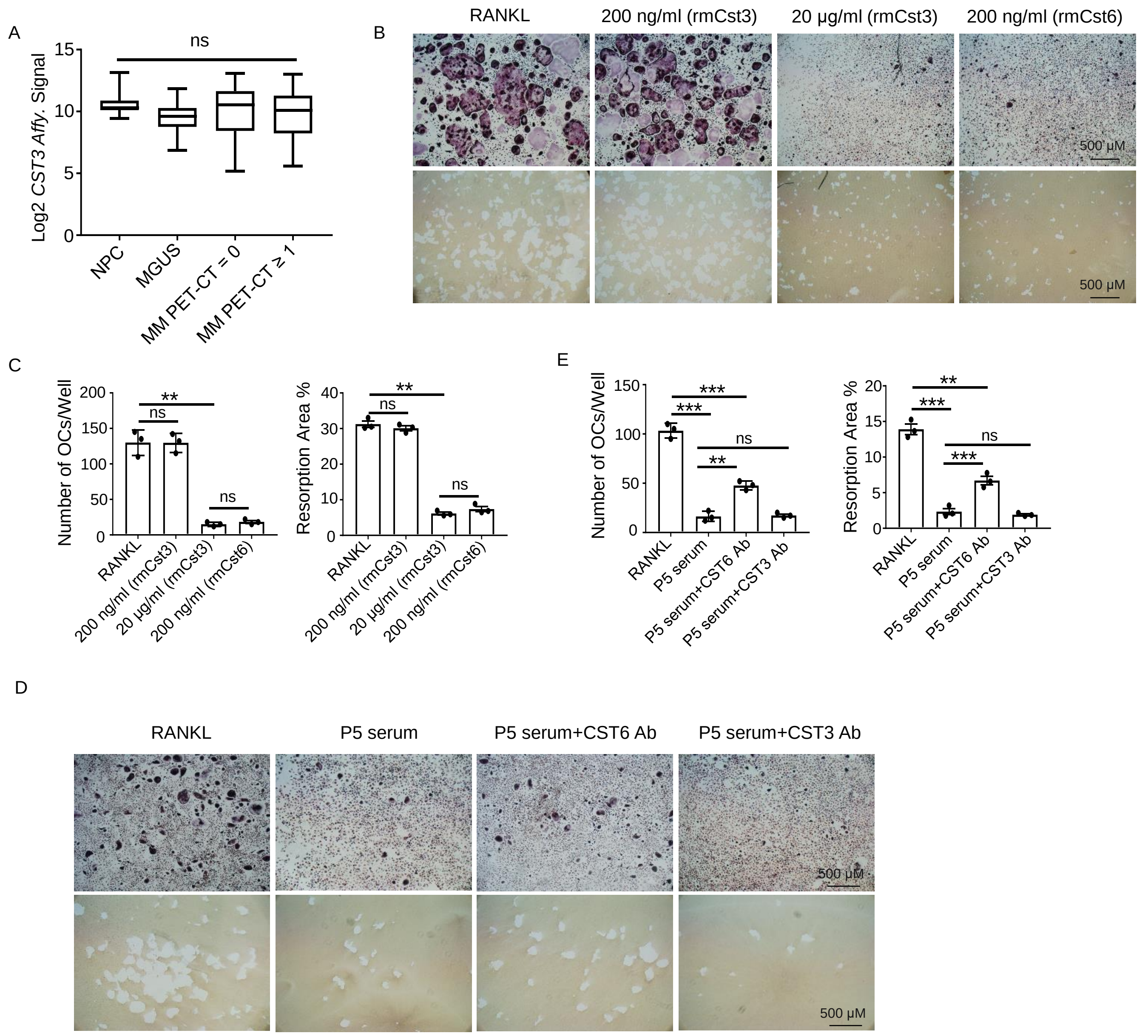

Figure S6. *CST3* expression in PC and CST3 protein function in OC differentiation and function. Related to Figure 3.
(A) A box plot shows the expression of *CST3* in NPC (n = 22), MGUS (n = 44) and MM patients with no PET-CT lesions (n = 178) or with 1 or more PET-CT lesions (n = 334). **(B)** Mouse BM monocytes (4 x 10^4 cells/well) were seeded into a 96-well plate and a Corning® Osteo Assay plate, TRAP staining and bone resorption assays were measured in mouse osteoclast precursors after culture in the presence of RANKL, +/- rmCst3 or rmCst6 for 4 days. **(C)** Bar-plots show the quantification of $TRAP^+$ osteoclast and the bone resorption area (n = 3). Scale Bar = 500 µm. **(D)** Human BM $CD14^+$ monocytes sorted from MM patient samples were differentiated into osteoclasts with M-CSF and RANKL. 10 µl BM serum from Patients 5 (P5) containing 200 ng/ml CST6 protein and 438 ng/ml CST3 protein was loaded into each well. 4 µg/ml of anti-CST6 antibody or 8 µg/ml of anti-CST3 antibody were also added to the culture media during human osteoclast differentiation. On day 14, half of the wells in each group were stained with TRAP solution and the remaining wells were quantified for resorption areas. The CST6 and CST3 level in each BM serum was determined by ELISA as described in Table S1. **(E)** Bar-plots show the quantification results of $TRAP^+$ osteoclasts and the bone resorption area (n = 3). Scale Bar = 500 µm. Data were represented as mean ± SEM and analyzed by one-way ANOVA with Tukey's multiple-comparisons (**A**, **C** and **E**). ***P* < 0.01, ****P* < 0.001; ns, not significant.

Figure S7

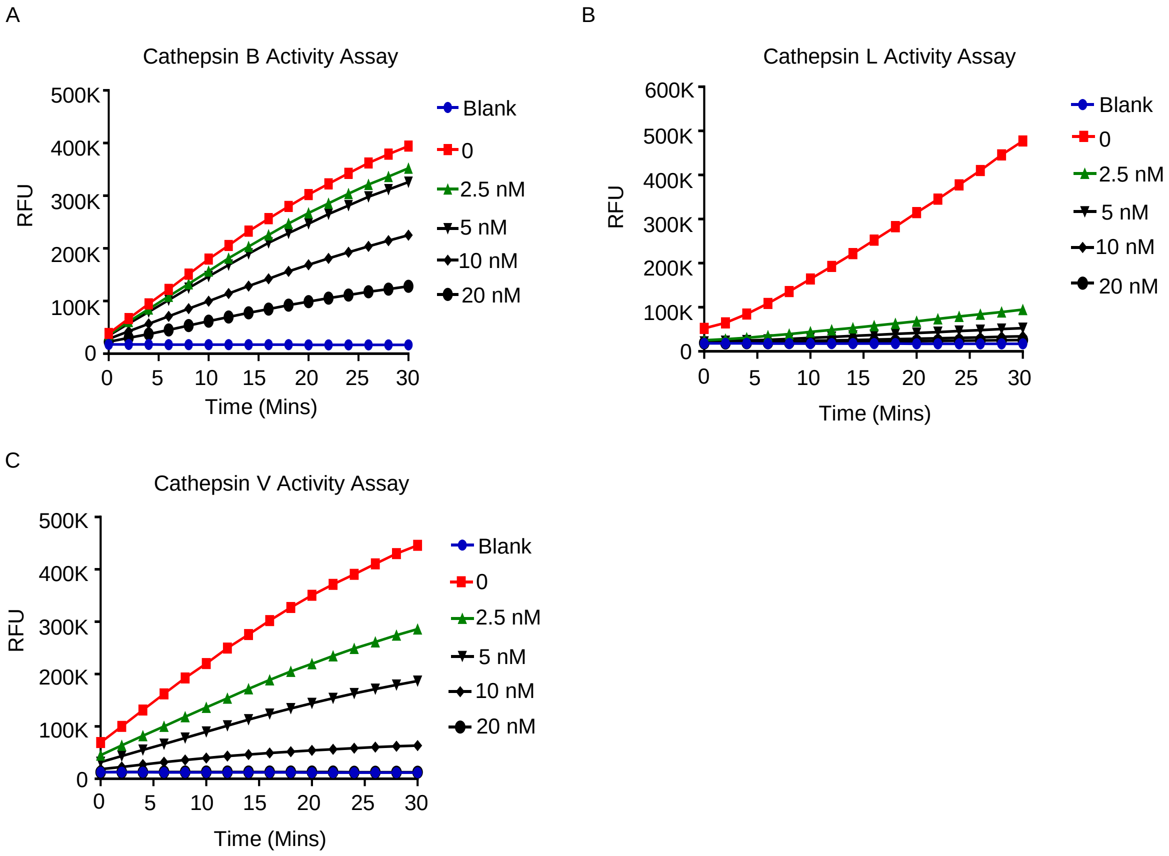

Figure S7. CST6 protein inhibits cathepsin B, L and V activity. Related to Figure 4.
(**A-C**) The cathepsin B, L, V activity were measured by the cathepsin B, L, V drug discovery kit (n = 3). The y axis represents the cathepsin B, L, V activity expressed as relative fluorescence units (RFU); the x axis is the time points treated by CST6 protein in multiple doses. Data are shown as mean ± SEM.

Figure S8

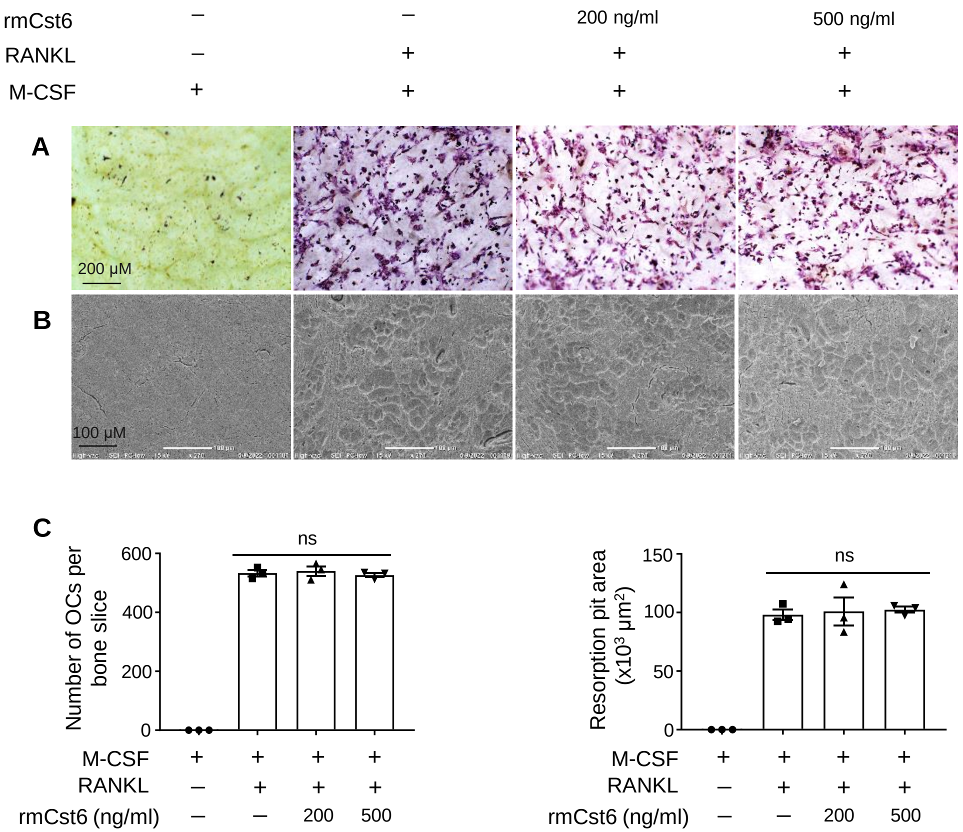

Figure S8. Low dose CST6 protein doesn't suppress the bone resorptive activity of mature osteoclasts. Related to Figure 5. (**A**) TRAP staining showed the mature osteoclasts on the bone slices (n = 3). Scale bar = 200μm. (**B**) After a 3-day culture period, osteoclasts were removed from bone slices, resorption pits were visualized by SEM, and resorption pit area was quantified (n = 3). Scale bar = 100μm. (**C**) Bar-plots show the quantification of $TRAP^+$ osteoclasts and the bone resorption area. Data represented as mean ± SEM. ns, not significant. Statistical analysis was performed using one-way ANOVA with Tukey's multiple-comparisons (**C**).

Figure S9

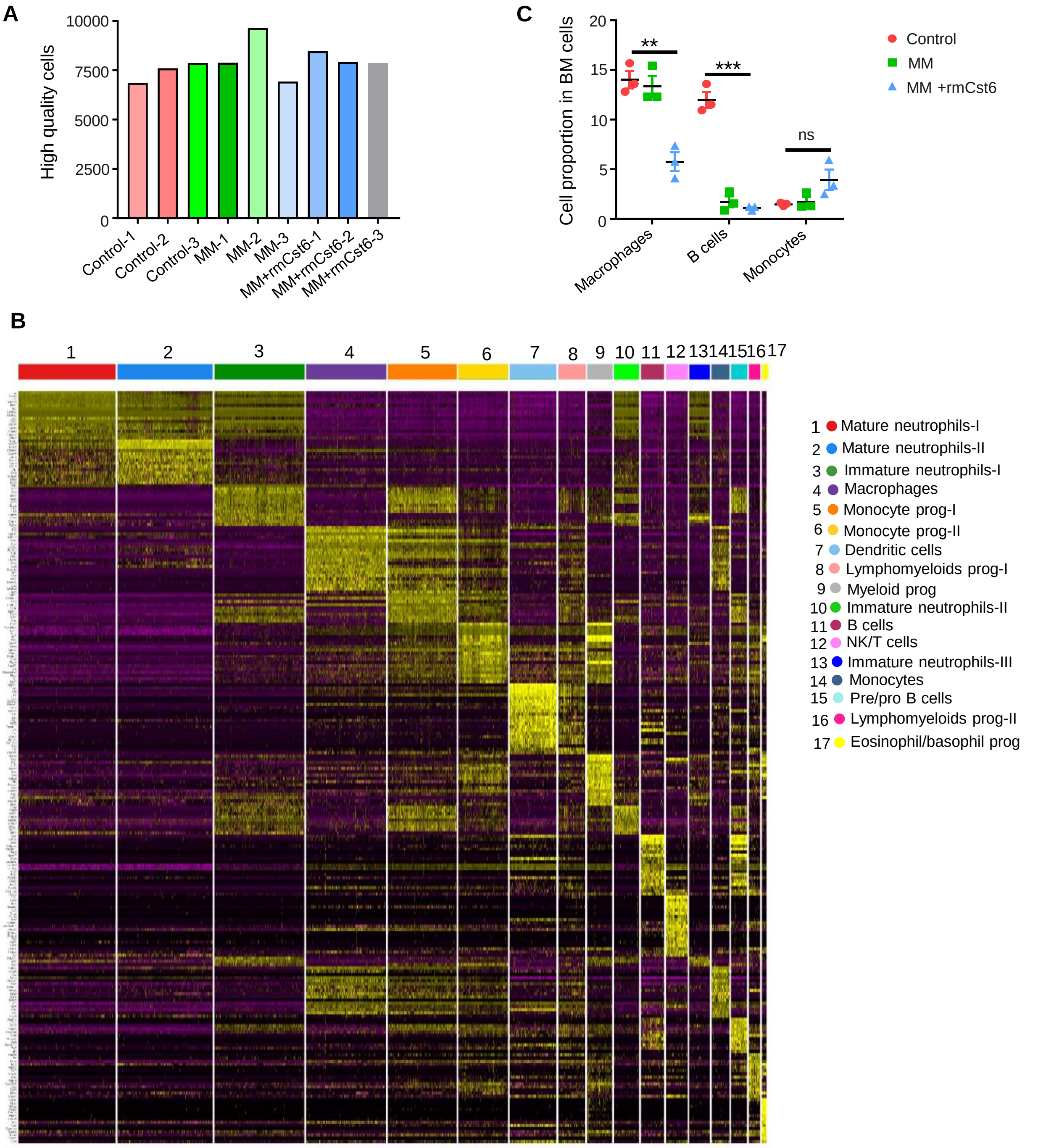

Figure S9. scRNA-seq identifies BM macrophage changes after treatment with CST6. Related to Figure 6.
(**A**) A bar-view shows the total cells sequenced in each group. (**B**) A heatmap showing the relative expression (log2 transformed) of marker genes across different immune cell types in mouse BM. (**C**) A bar-view shows subpopulation of macrophage, B cells and monocytes in each group. Data are represented as mean ± SEM. ***P* < 0.01, ****P* < 0.001. ns, not significant. Statistical analysis were performed using one-way ANOVA with Tukey's multiple-comparisons (**C**).

Figure S10

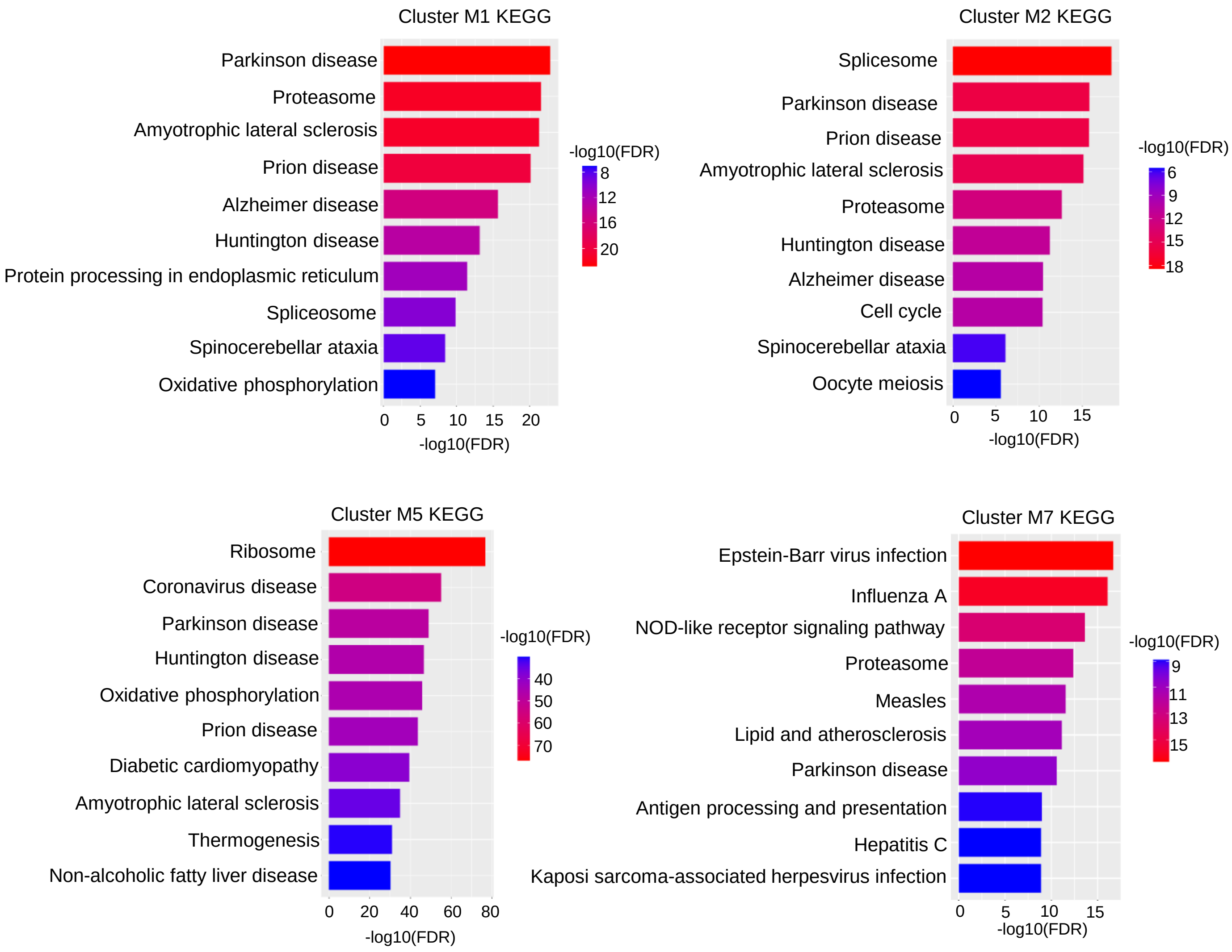

Figure S10. KEGG pathway analyses of genes upregulated in each cluster. Related to Figure 6.
Bar-views showed the KEGG pathway analysis of upregulated genes in each cluster (M1, M2, M5 and M7).

Figure S11

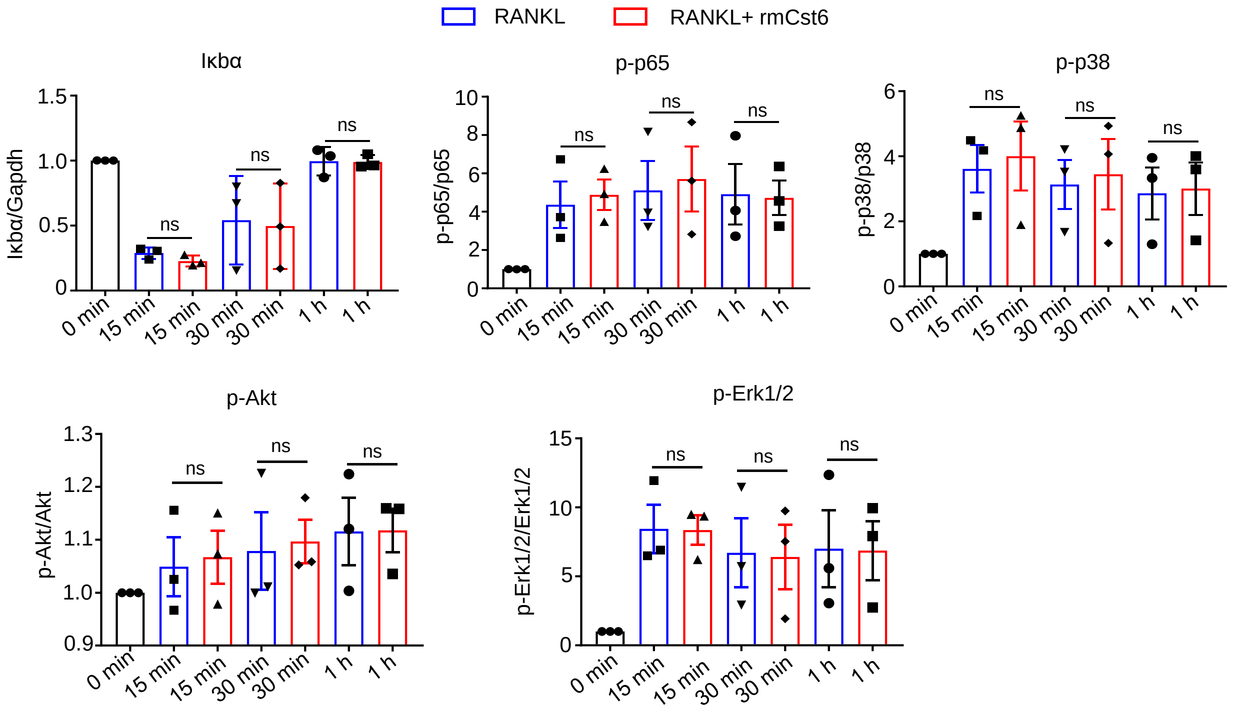

Figure S11. CST6 protein selectively inhibits the non-canonical NF-κB signaling pathway induced by RANKL. Related to Figure 7. Bar-plots showed the quantification of the ratios of band intensity relative to Iκbα, p-p65, p-p38, p-Akt and p-Erk1/2 (n = 3). Data are represented as mean ± SEM. ns, not significant. Statistical analysis was performed using unpaired, 2-sided, independent Student's t test.

Figure S12

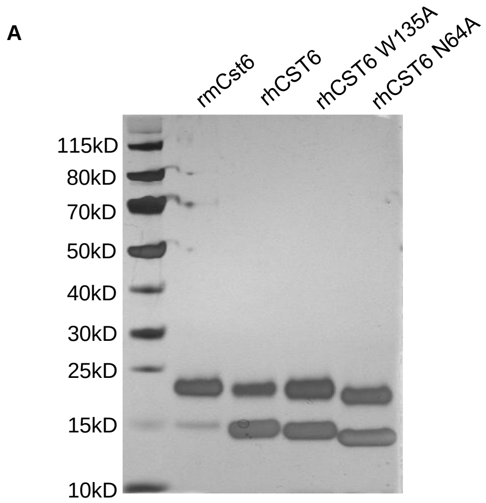

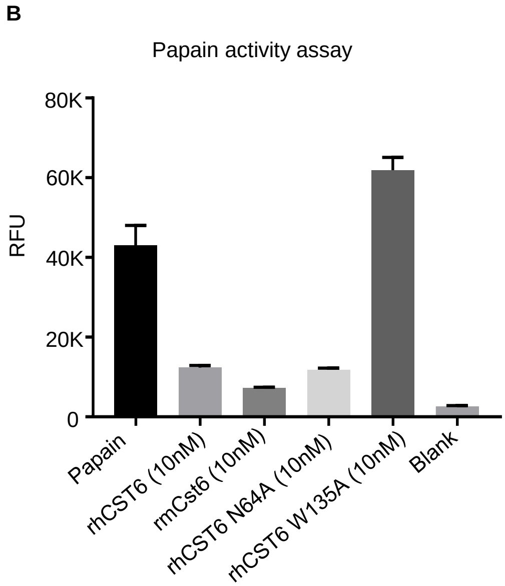

Figure S12. Purification of WT and mutant CST6 proteins and enzymatic activity assay. Related to Figure 8.
(**A**) Silver staining of WT and mutant CST6 proteins after over-expression and purification (n = 3). (**B**) A bar-view showed that the Papain activity was suppressed by WT and N64A CST6 proteins but not by W135A CST6 protein (n = 3).

Table S11. Oligonucleotides used in the study.	
For mutagenesis	
CST6 W135A variant F	5′-GGTCCTTGTGGTTCCCGCGCAGAACTCCTCTCAGC-3'
CST6 W135A variant R	5′-GCTGAGAGGAGTTCTGCGCGGGAACCACAAGGACC-3′
CST6 N64A variant F	5′-GCTACAACATGGGCAGCGCCAGCATCTACTACTTCC-3′
CST6 N64A variant R	5′-GGAAGTAGTAGATGCTGGCGCTGCCCATGTTGTAGC-3′

Figure 7A

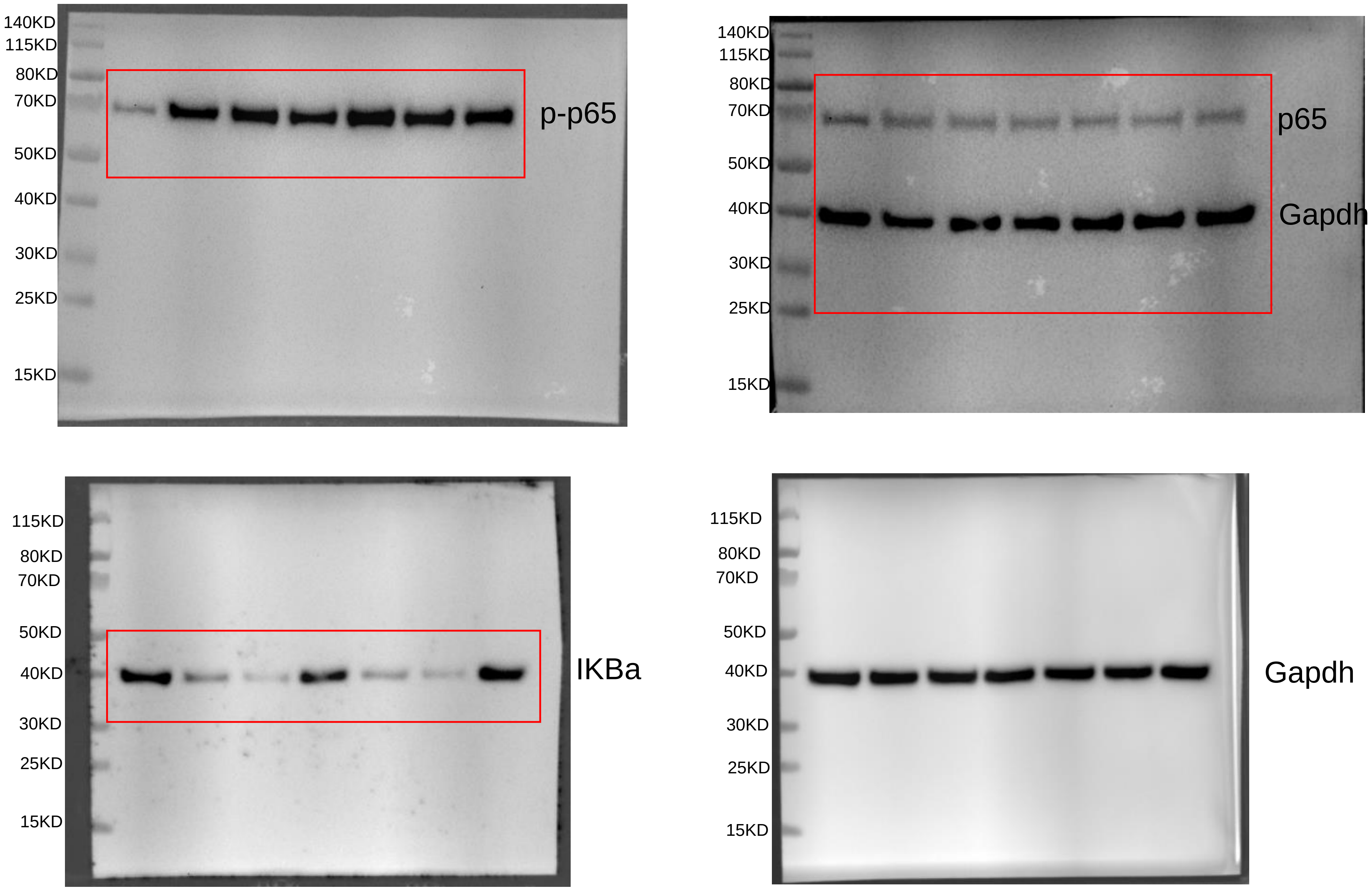
140KD
115KD
80KD
70KD
50KD
40KD
30KD
25KD
15KD
p-p65
p65
Gapdh
IKBa
Gapdh

Figure 7A

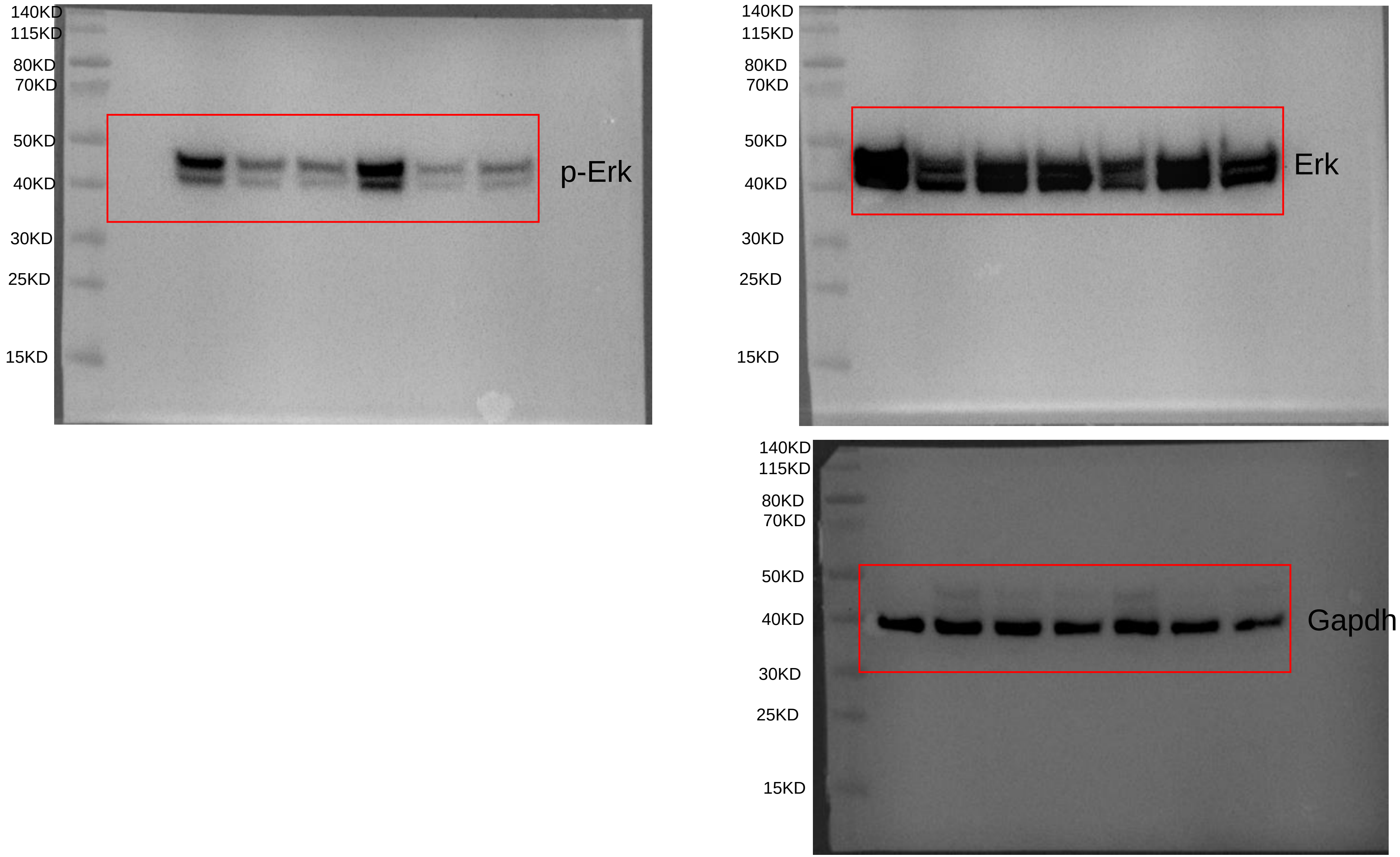
140KD
115KD
80KD
70KD
50KD
40KD
30KD
25KD
15KD
p-Erk
Erk
Gapdh

Figure 7A

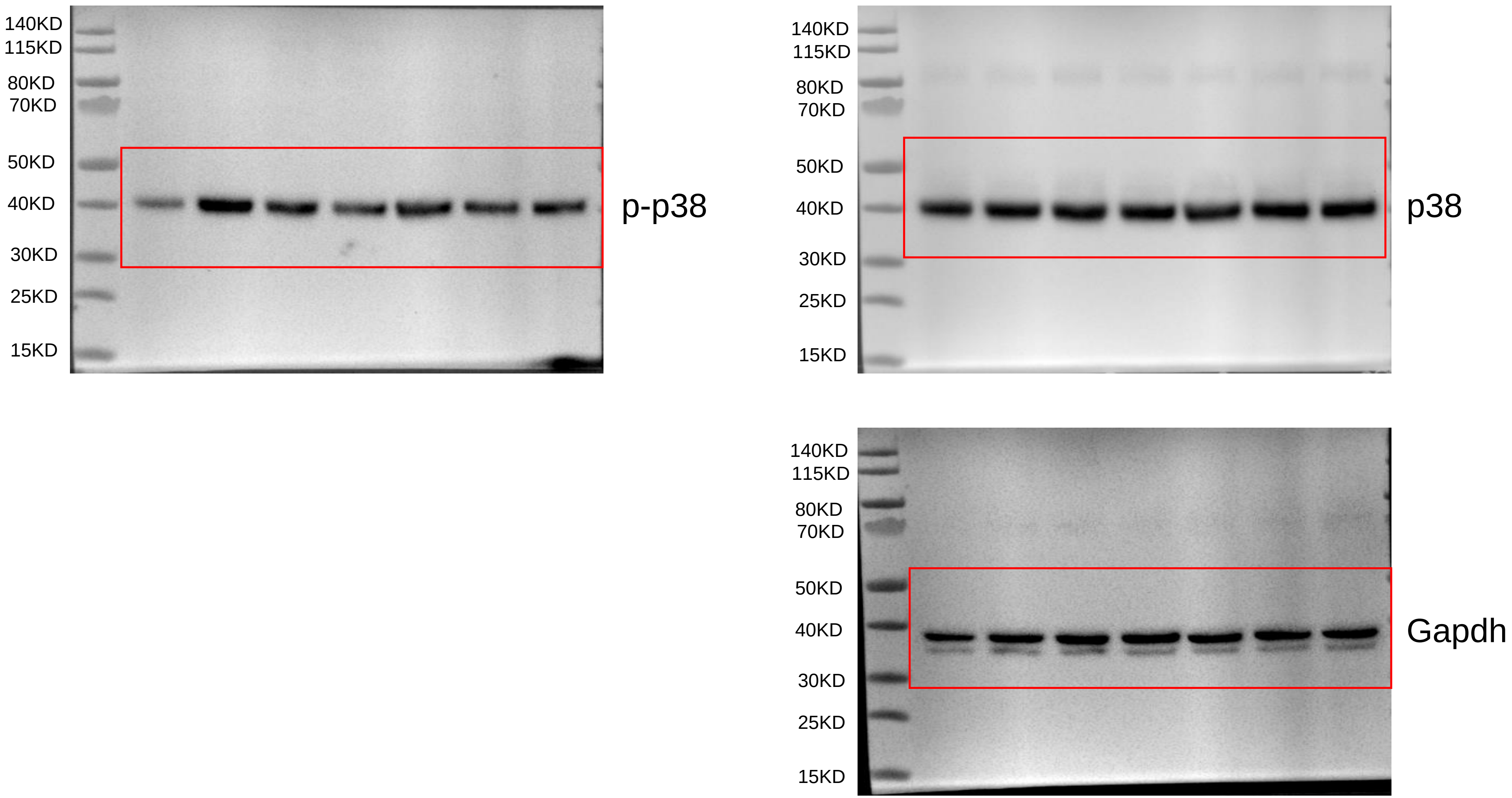
140KD
115KD
80KD
70KD
50KD
40KD
30KD
25KD
15KD
p-p38
140KD
115KD
80KD
70KD
50KD
40KD
30KD
25KD
15KD
p38
140KD
115KD
80KD
70KD
50KD
40KD
30KD
25KD
15KD
Gapdh

Figure 7A

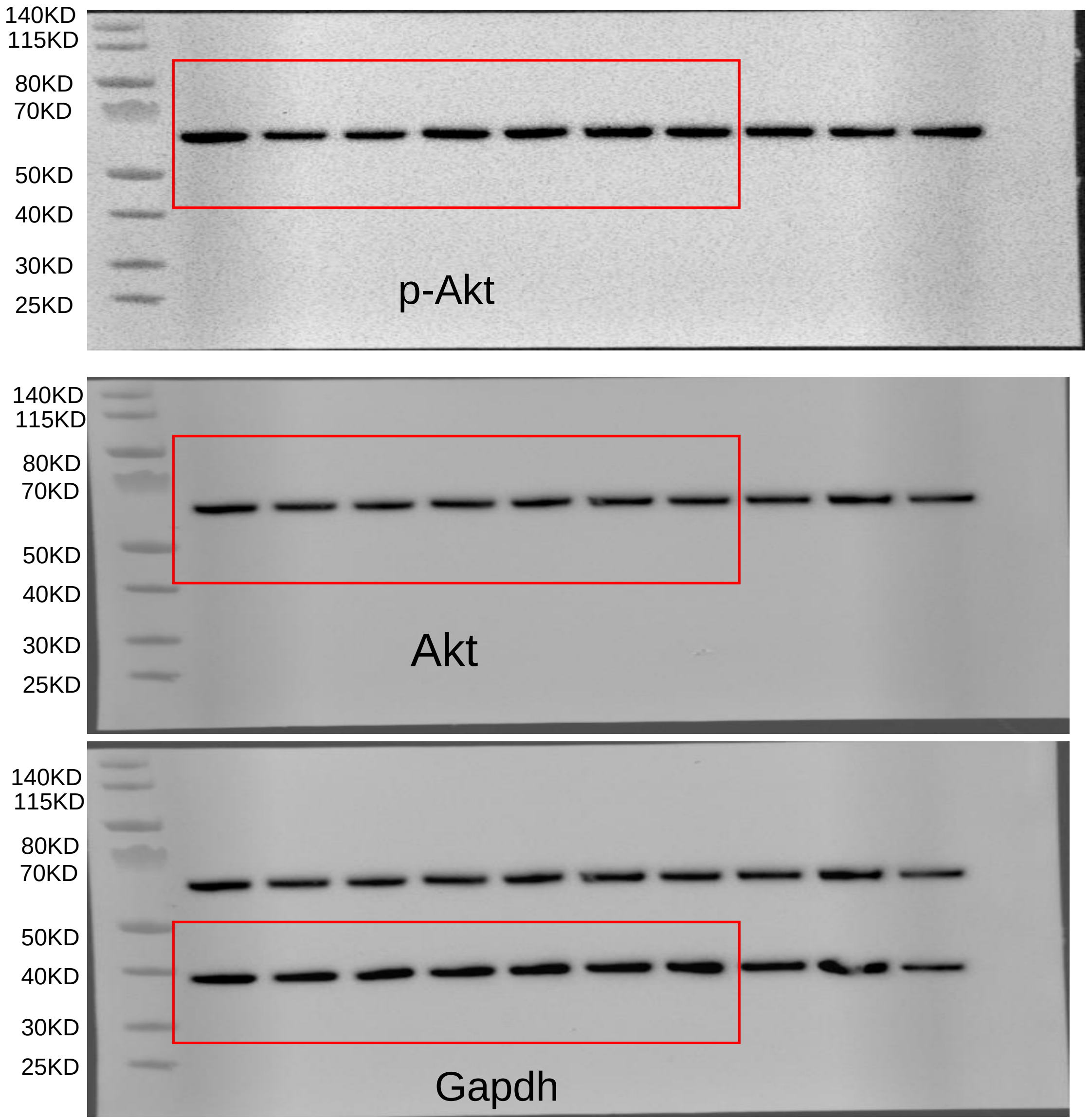
140KD
115KD
80KD
70KD
50KD
40KD
30KD
25KD
p-Akt
140KD
115KD
80KD
70KD
50KD
40KD
30KD
25KD
Akt
140KD
115KD
80KD
70KD
50KD
40KD
30KD
25KD
Gapdh

Figure 7B

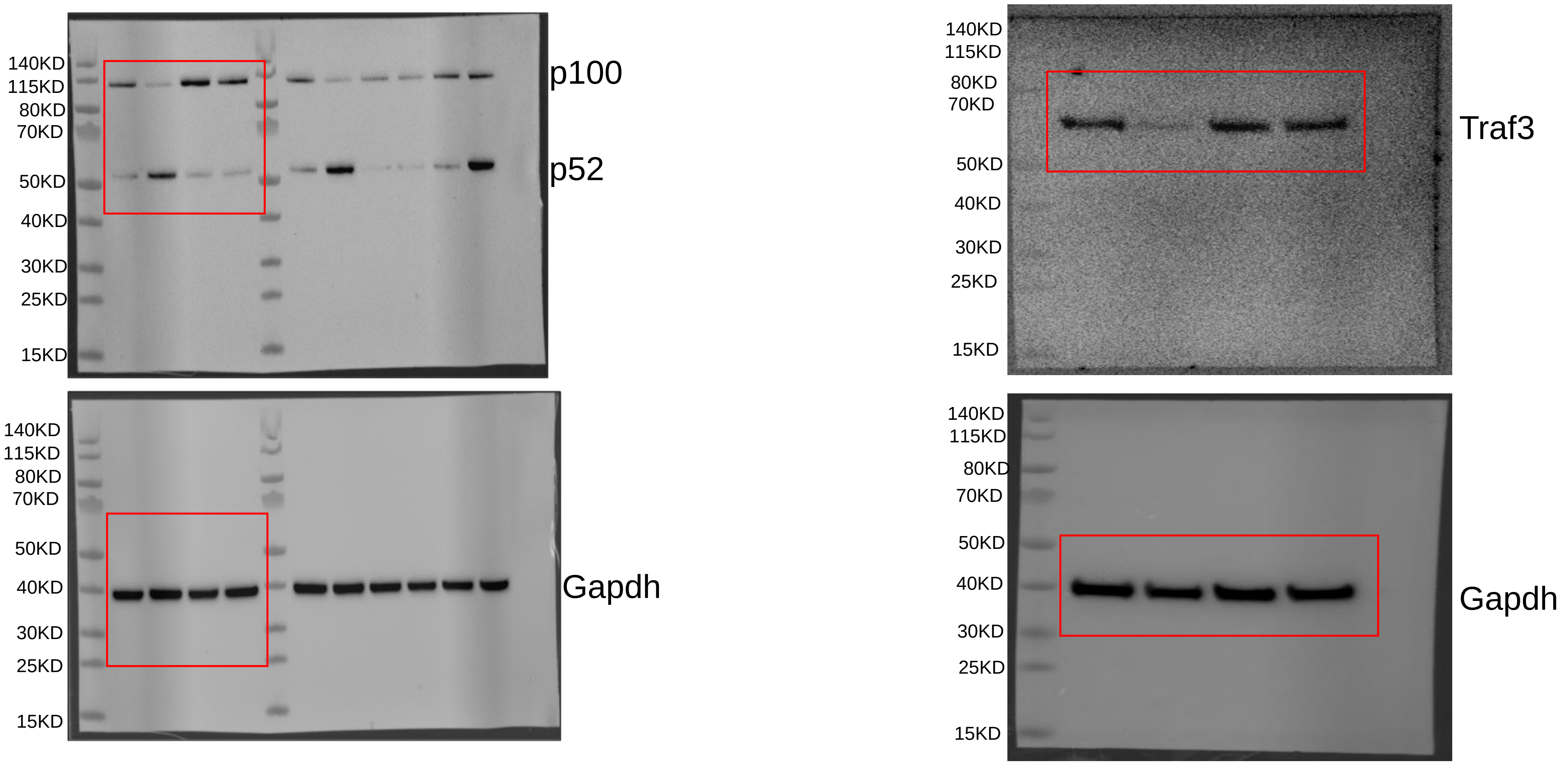
140KD
115KD
80KD
70KD
50KD
40KD
30KD
25KD
15KD
p100
p52
140KD
115KD
80KD
70KD
50KD
40KD
30KD
25KD
15KD
Gapdh
140KD
115KD
80KD
70KD
50KD
40KD
30KD
25KD
15KD
Traf3
140KD
115KD
80KD
70KD
50KD
40KD
30KD
25KD
15KD
Gapdh

Figure 7F

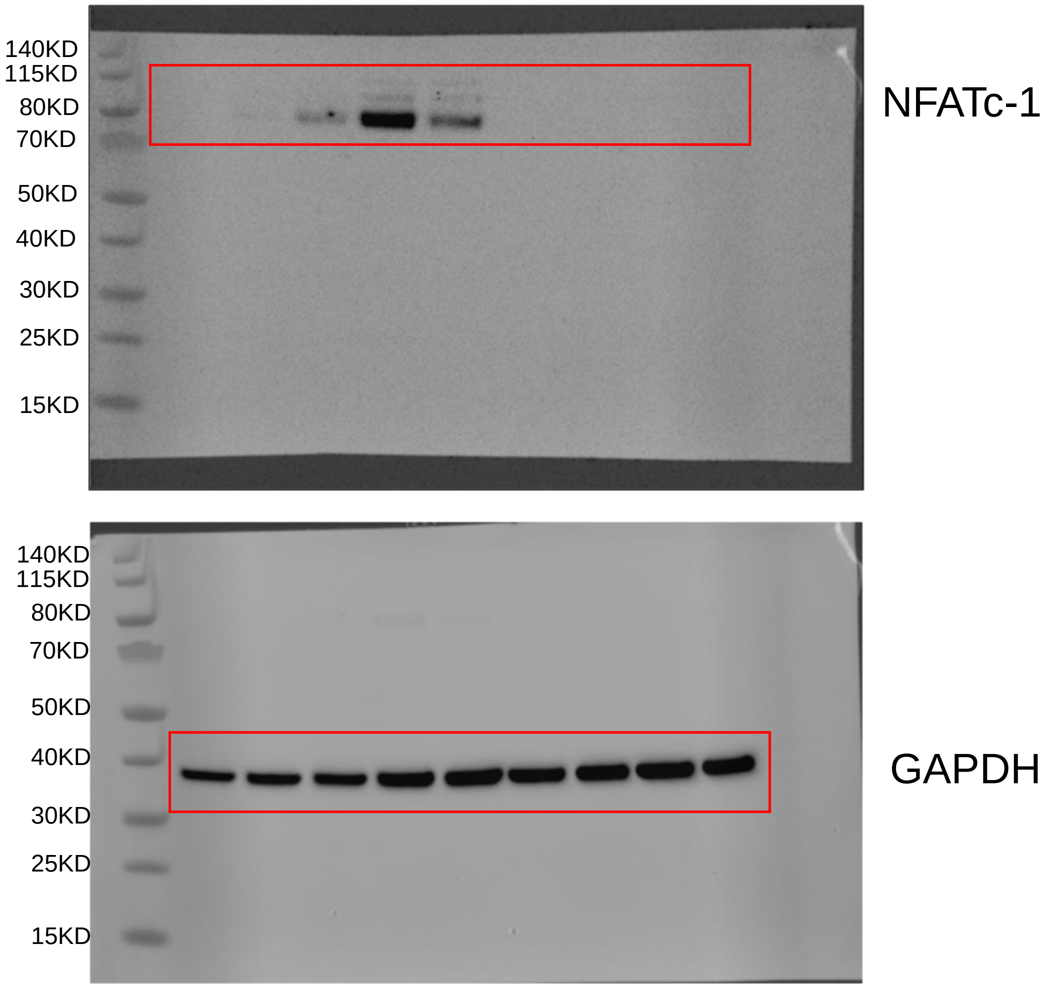
140KD
115KD
80KD
70KD
50KD
40KD
30KD
25KD
15KD
NFATc-1
140KD
115KD
80KD
70KD
50KD
40KD
30KD
25KD
15KD
GAPDH

Figure 7F

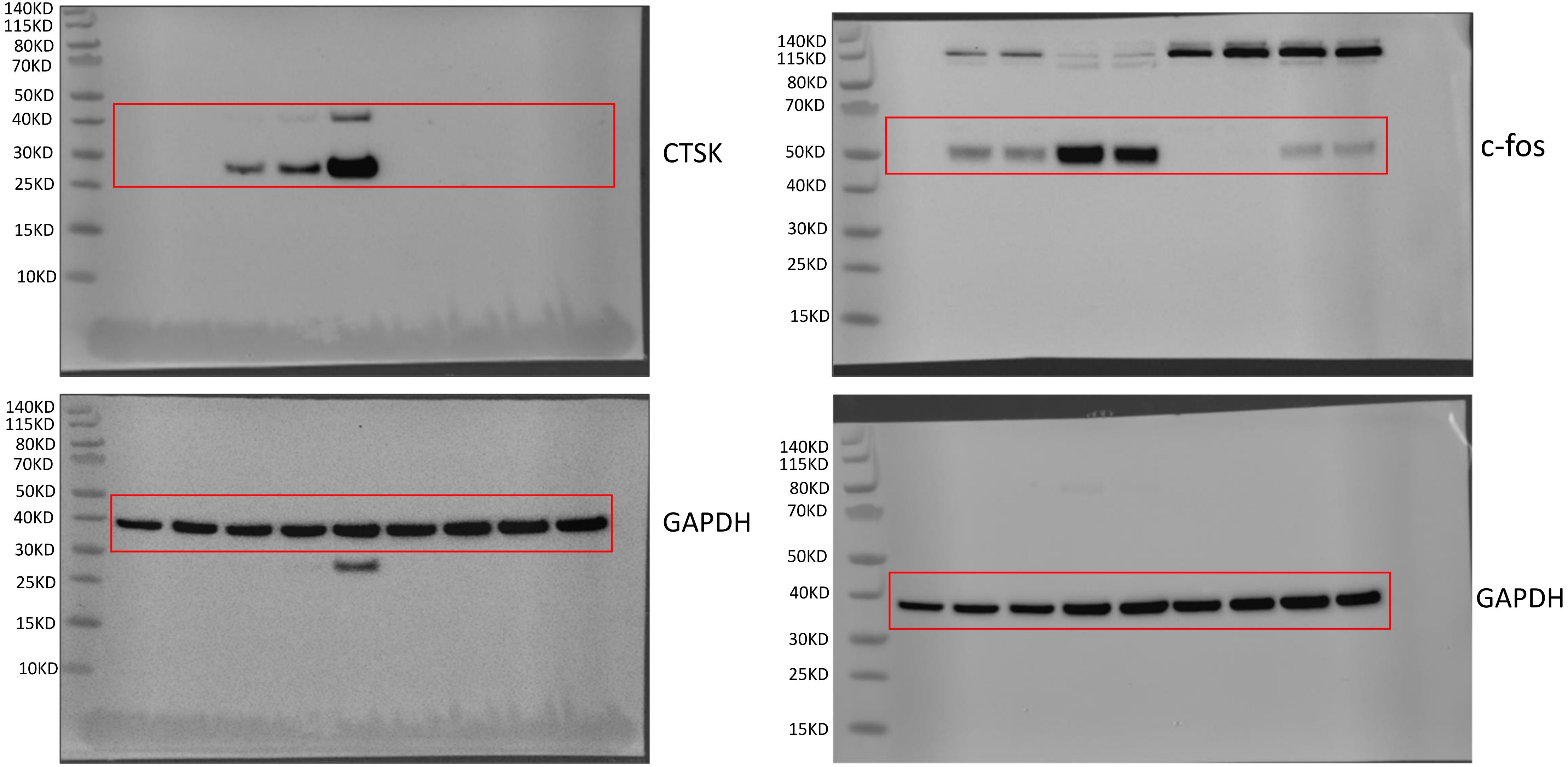
140KD
115KD
80KD
70KD
50KD
40KD
30KD
25KD
15KD
10KD
CTSK
c-fos
GAPDH
GAPDH

Figure 8A

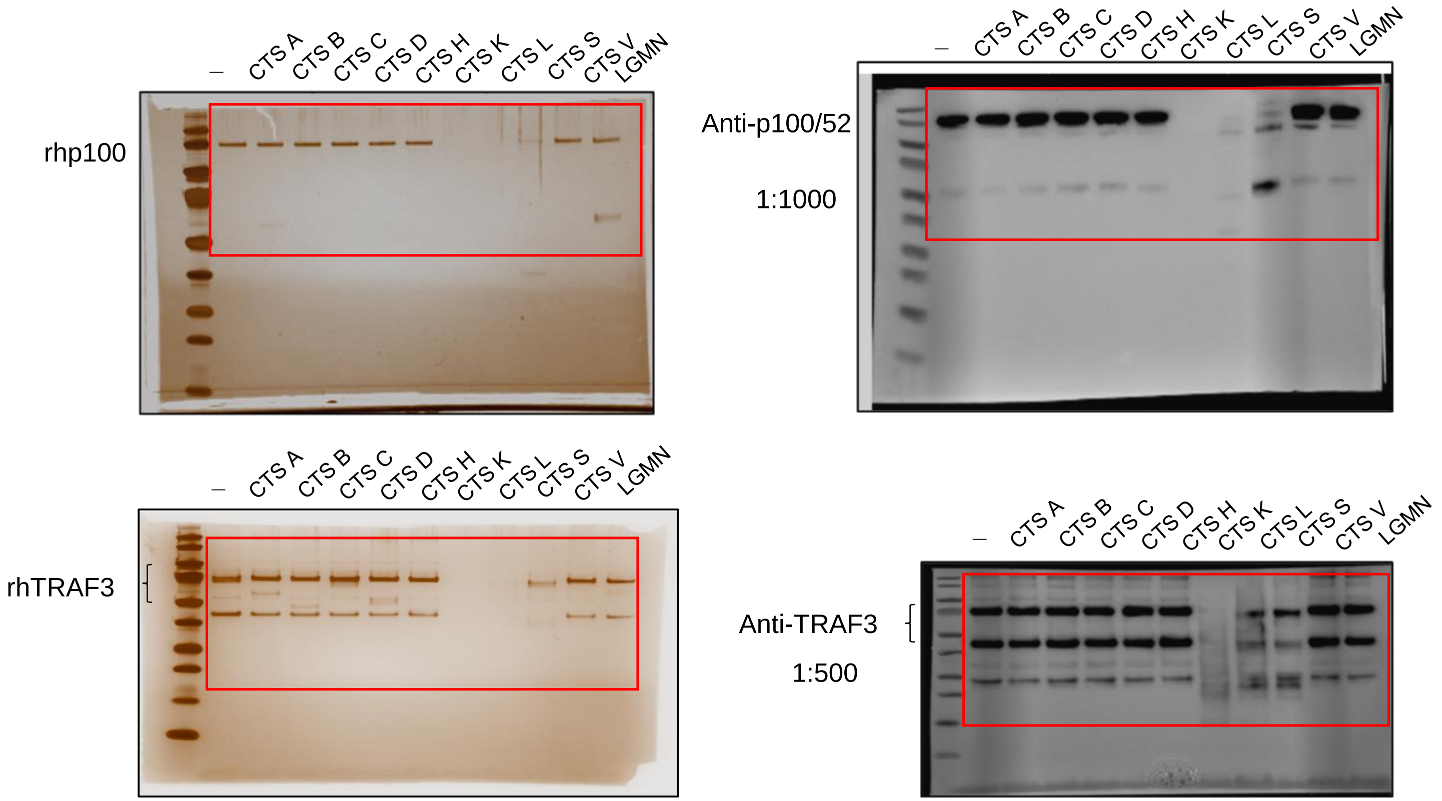

–
CTS A
CTS B
CTS C
CTS D
CTS H
CTS K
CTS L
CTS S
CTS V
LGMN
rhp100
Anti-p100/52
1:1000
rhTRAF3
Anti-TRAF3
1:500

Figure 8D

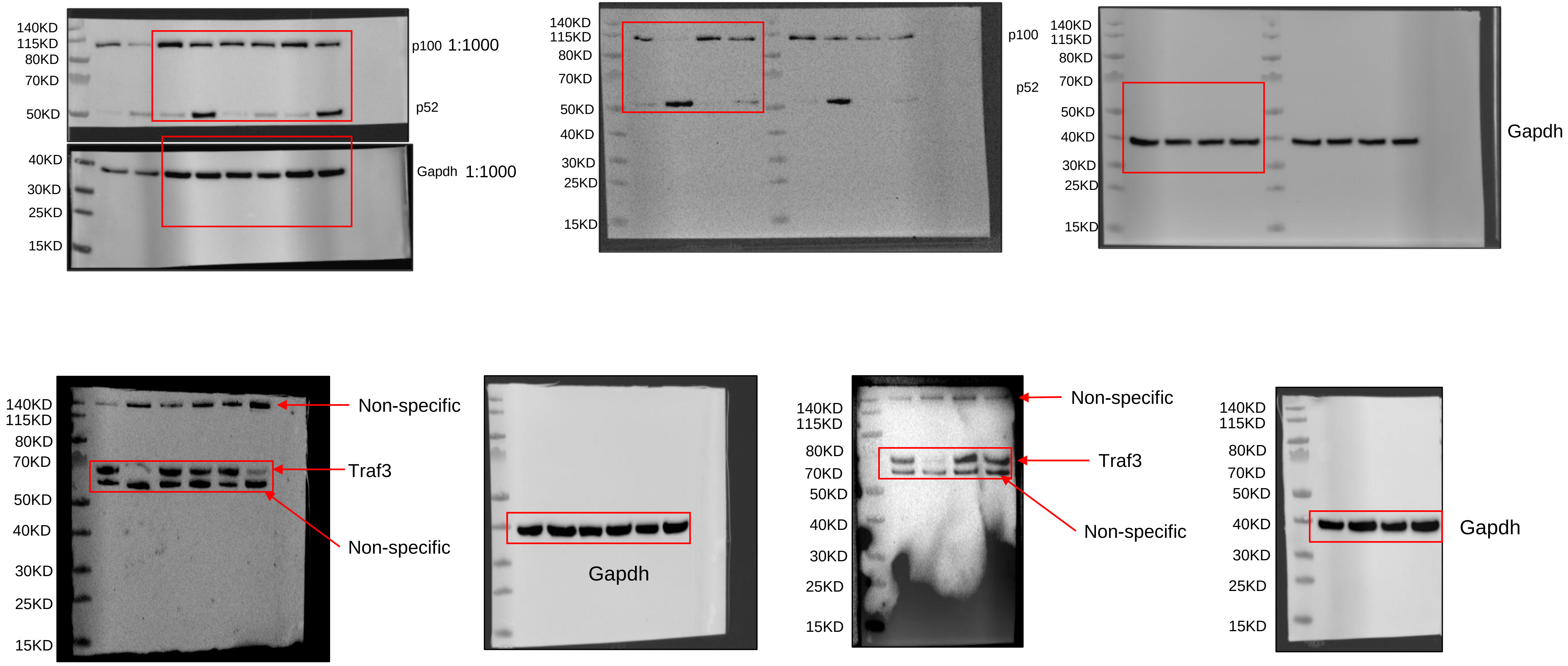
140KD
115KD
80KD
70KD
50KD
40KD
30KD
25KD
15KD
p100 1:1000
p52
Gapdh 1:1000
p100
p52
Gapdh
Non-specific
Traf3
Non-specific
Gapdh
Non-specific
Traf3
Non-specific
Gapdh

Figure 8E

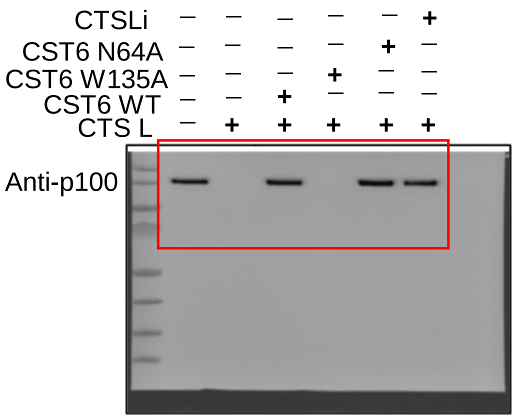
CTSLi
CST6 N64A
CST6 W135A
CST6 WT
CTS L
Anti-p100

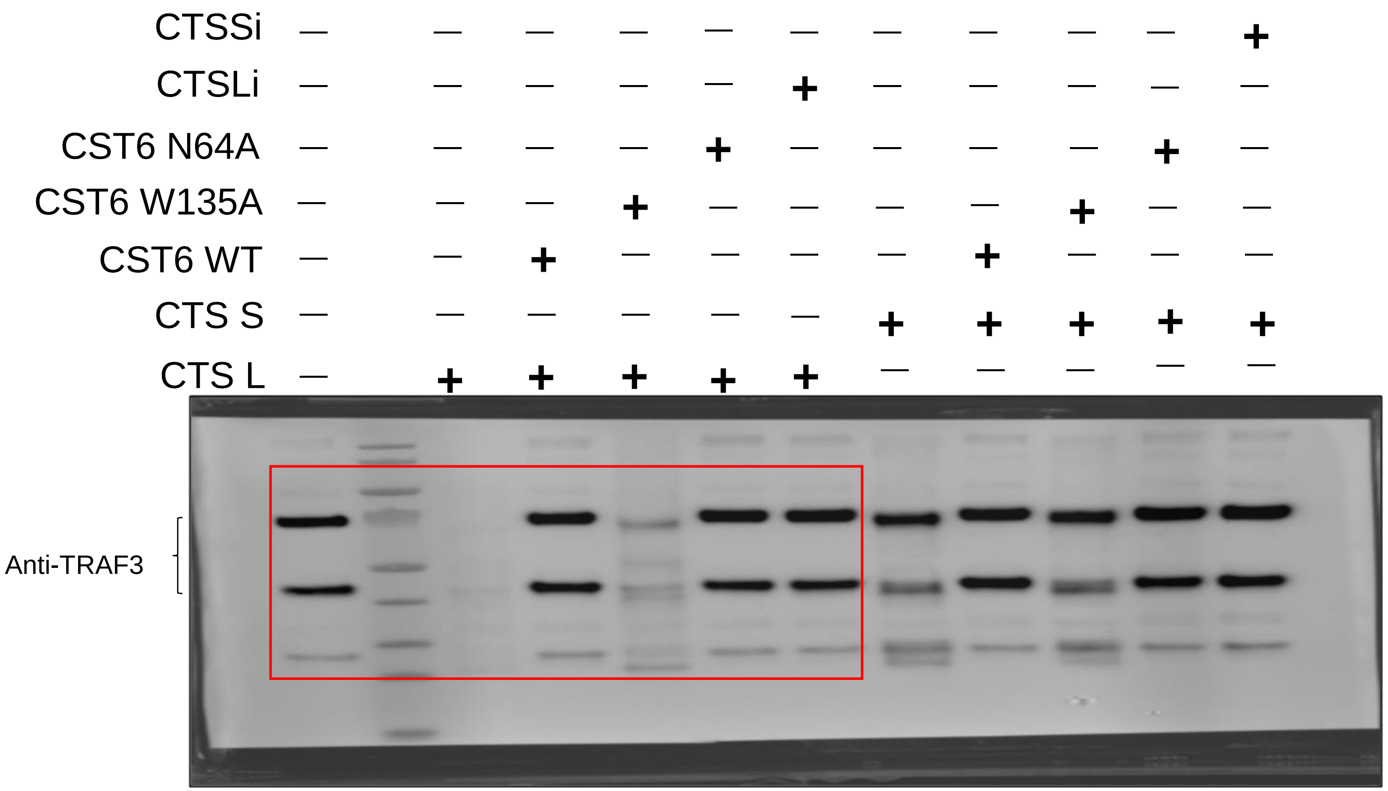
CTSSi
CTSLi
CST6 N64A
CST6 W135A
CST6 WT
CTS S
CTS L
Anti-TRAF3

Figure 8H

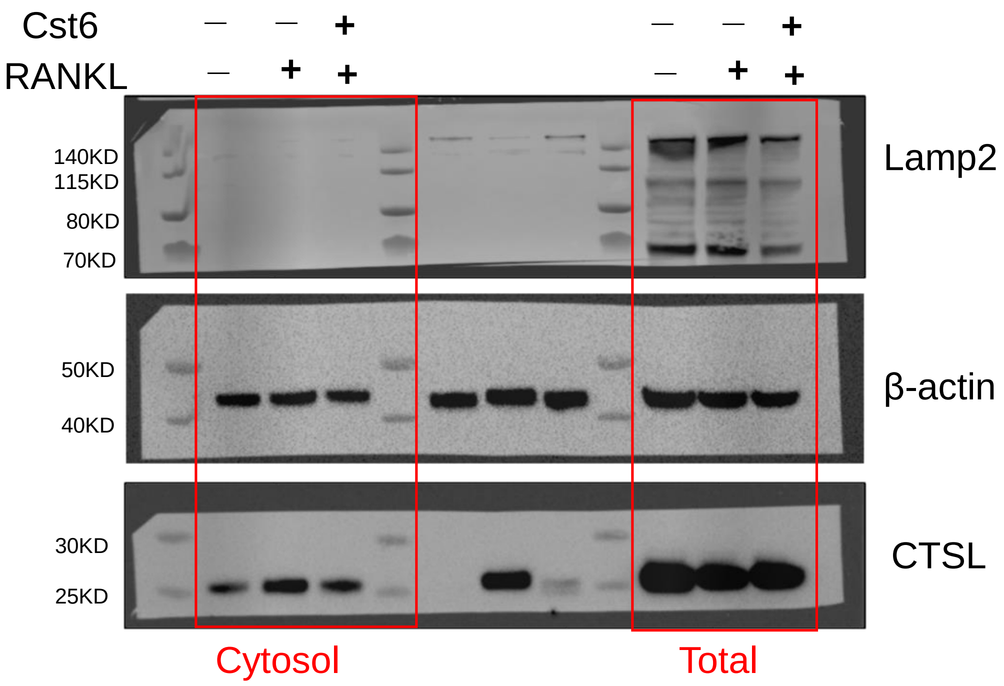

Cst6
RANKL
—
—
+
—
+
+
—
—
+
—
+
+
140KD
115KD
80KD
70KD
Lamp2
50KD
40KD
β-actin
30KD
25KD
CTSL
Cytosol
Total
